# Olink Proteomics Reveals CCL2 Aggravates Perihematomal Edema After Intracerebral Hemorrhage in High‐Altitude Migrants Via CCR2/NF‐κB‐Mediated Blood–Brain Barrier Disruption

**DOI:** 10.1002/cns.70868

**Published:** 2026-04-07

**Authors:** Rongsu Huang, Ling Gao, Yalan Dai, Shangshi Li, Jiancai Guru, Mingxi Li, Yù‐Jié Chen, Bo Wu, Xufang Ru, Qing Gong, Jun Tang, Gang Zhu, Yujie Chen

**Affiliations:** ^1^ Department of Neurosurgery, Southwest Hospital Third Military Medical University (Army Medical University) Chongqing China; ^2^ Department of Neurosurgery General Hospital of Tibet Military Command Lhasa China; ^3^ Chongqing Key Laboratory of Intelligent Diagnosis, Treatment and Rehabilitation of Central Nervous System Injuries, Southwest Hospital Third Military Medical University (Army Medical University) Chongqing China; ^4^ Chongqing Clinical Research Center for Neurosurgery, Southwest Hospital Third Military Medical University (Army Medical University) Chongqing China; ^5^ Department of Information, Southwest Hospital Third Military Medical University (Army Medical University) Chongqing China; ^6^ Department of Neurosurgery Children's Hospital of Chongqing Medical University Chongqing China; ^7^ Department of Mountain Disease General Hospital of Tibet Military Command Lhasa China; ^8^ Department of Neurosurgery, Chongqing General Hospital Chongqing University School of Medicine Chongqing China

**Keywords:** blood–brain barrier, CCL2, high altitude, intracerebral hemorrhage, perihematomal edema

## Abstract

**Aims:**

To identify key environment‐sensitive inflammatory factors and clarify their pathogenic mechanisms in severe secondary brain injury after intracerebral hemorrhage (ICH) in high‐altitude migrants—addressing the critical gap that elevated hemoglobin alone cannot fully explain the underlying pathogenesis and investigating chronic inflammation as a key pathogenic driver.

**Methods:**

Olink proteomics profiling was performed in 66 high‐altitude migrants and 22 plain‐resident controls to identify differentially expressed inflammatory factors. Rat high‐altitude ICH models were subsequently established and treated with AAV‐CCL2, a CCL2‐neutralizing antibody, recombinant CCL2, and a CCR2 inhibitor, and the results were validated through transcriptomic and multidimensional methods.

**Results:**

CCL2 was the most significantly upregulated environmentally sensitive inflammatory factor and was weakly correlated with hemoglobin levels. High‐altitude rats had higher levels of post‐ICH CCL2, which was associated with severe blood–brain barrier (BBB) disruption; CCR2 blockade abolished CCL2‐induced BBB damage and neuroinflammation.

**Conclusions:**

CCL2 plays an important role in secondary injury after high‐altitude ICH, mainly by amplifying inflammation and exacerbating damage via the CCL2–CCR2–NF‐κB pathway and may serve as a potential therapeutic target.

## Graphical abstract

Using high‐altitude migrants and rat models, we demonstrated CCL2 is a key inflammatory factor exacerbating high‐altitude ICH injury: long‐term high‐altitude exposure increases baseline CCL2 levels, and ICH triggers a sharp increase in CCL2 levels, which drives inflammatory storm amplification, BBB disruption, and neurological deficits. Inhibiting CCL2/CCR2/NF‐κB pathway mitigates high‐altitude post‐ICH injury, offering a novel target for clinical prevention and treatment of high‐altitude ICH.

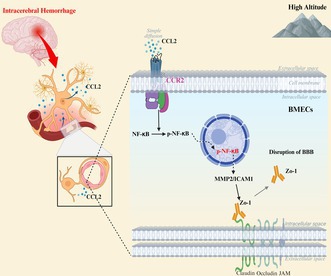

**Abbreviations**
AAV: adeno‐associated virus
AAV‐CCL2‐INT: AAV vector expressing shRNA targeting CCL2
BBB: blood–brain barrier
BSA: bovine serum albumin
BWC: brain water content
CCL2‐Ab: CCL2 neutralizing antibody
CCR2‐I: CCR2‐specific inhibitor
CMS: chronic mountain sickness
DEGs: differentially expressed genes
DMSO: dimethyl sulfoxide
EB: evans blue
ELISA: enzyme‐linked immunosorbent assay
GO: gene ontology
GSEA: gene set enrichment analysis
HA: high‐altitude adaptation population group
HACE: high‐altitude cerebral edema
HAPE: high‐altitude pulmonary edema
HAPH: high‐altitude pulmonary hypertension
HH: high‐altitude hyperhemoglobinemia group
HS: high‐altitude subclinical polycythemia group
ICH: intracerebral hemorrhage
ICU: intensive care unit
IF: immunofluorescence
KEGG: kyoto encyclopedia of genes and genomes
MNSS: modified neurological severity score
MRI: magnetic resonance imaging
MWM: morris water maze
PBS: phosphate‐buffered saline
PC: plain‐resident control group
PEA: proximity extension assay
R‐CCL2: recombinant CCL2 protein
TEM: transmission electron microscopy
WB: western blot

## 1 Introduction

High‐altitude migrants are individuals who have been born/residing long term in lowlands (≤ 1000m) and who migrate to high‐altitude regions (≥ 3000m) for work/living, etc., with more than 6 months of residence. Their numbers are increasing [1], and chronic high‐altitude exposure increases the risk of intracerebral hemorrhage [2], with higher severity, mortality, and disability [3, 4, 5, 6, 7, 8, 9] in comparison with those in lowlanders and high‐altitude natives [10]. In Tibet, ICH accounts for 49.5% of strokes (national average of 14.2%) [7], with migrants presenting earlier onset [5], larger hematomas, and poorer outcomes [6]. Hypoxia and elevated hemoglobin (Hb) [8, 11] are proposed contributors; however, high‐altitude migrants suffer from severe postintracerebral hemorrhage (ICH) injury following early hematoma evacuation and adequate oxygen supplementation—indicating mechanisms that cannot be fully explained by elevated Hb alone. Chronic hypoxia induces inflammatory factor overexpression [12, 13], driving acute high‐altitude edema [10, 14, 15] and chronic cerebral decline [14, 16, 17], whereas inflammatory “sensitization” [18] may reshape the cerebral microenvironment to exacerbate secondary ICH injury. However, key inflammatory pathways and mechanisms remain unclear, with prior studies limited by narrow inflammatory coverage and small sample sizes [13]. Thus, this study aimed to screen potential inflammatory factors and clarify their roles in secondary high‐altitude ICH injury.

## 2 Materials and Methods

### 2.1 Clinical Sample Collection and Processing

This study was approved by the Ethics Committee of the General Hospital of Tibet Military Command (Approval No. 24‐Ke‐007‐01) and was conducted by the Declaration of Helsinki, with informed consent obtained from all participants (Figure S1, Experiment 1). A total of 99 male high‐altitude migrants (≥ 3000m, residence ≥ 1 year; H‐group: mean altitude 4173 m, exposure 8.99 years) and 25 male plain‐resident controls (< 500m; PC group) were included (mean age 29 years). The H‐group was subdivided by hemoglobin (Hb) and CMS scoring [19]. HH (high‐altitude hyperhemoglobinemia, Hb > 210 g/L), HS (high‐altitude subclinical polycythemia, 180–210 g/L), HA (high‐altitude adaptation, 120–180 g/L), and PC (plain control, Hb 120–180 g/L). A total of 27 high‐altitude migrant volunteers were excluded from the study because of a history of infection or trauma, whereas another 6 were excluded because of abnormally elevated white blood cell counts detected by routine hematological examination. Ultimately, 22 participants were assigned to each subgroup within the H groups. From 25 eligible plain volunteers, 22 were randomly selected for sampling, with all the results meeting the requirements (details in Table S1 and Table S2). Peripheral blood was collected in EDTA tubes; plasma was separated by centrifugation and stored at −80°C.

### 2.2 Olink Proteomics Analysis

The Explore Inflammation Panel from Olink (Uppsala, Sweden) was used to quantify the expression of 92 inflammation‐related proteins in each patient [20]. Proximity extension assay (PEA) technology was used for the Olink protocol, which enables 92 analytes to be analyzed simultaneously. The resulting DNA sequence was subsequently detected and quantified using a microfluidic real‐time PCR instrument (Signature Q100, LC‐Bio Technology Co. Ltd., Hangzhou, China). Data analysis was performed using R software (v4.2.1). Pairwise comparisons between the three high‐altitude subgroups and the plain control group, as well as between the overall high‐altitude group and plain control, were conducted using independent‐sample *t*‐tests (*p* < 0.05). A Venn diagram was used to identify core differentially expressed proteins (DEPs). To explore their biological implications, Gene Ontology (GO) and Kyoto Encyclopedia of Genes and Genomes (KEGG) enrichment analyses were performed (*p* < 0.05).

### 2.3 Experimental Animals

All experiments in this study were approved by the Laboratory Animal Welfare and Ethics Committee of the Army Medical University (Approval No. AMUWEC20232224) and reported in accordance with the ARRIVE 2.0 guidelines. To better simulate individuals migrating to high‐altitude areas, Sprague–Dawley rats, characterized by poor adaptability and severe adaptive responses to high altitude, were selected as the experimental animals in this study [21, 22, 23]. To avoid the effects of the estrogen cycle on ICH and the high‐altitude model, 519 SPF male SD rats, weighing 220–250 g, were used. A schematic diagram of the experimental design is provided in Figure S1 and details are listed Table S3. The animals were euthanized upon completion of the experiment.

### 2.4 Chronic High‐Altitude Exposure and ICH Rat Models

Combining prior methods with our hospital's small‐animal hypobaric chamber, we optimized a chronic high‐altitude exposure rat model via iterative pilot experiments [24] (Figure S1, Experiments 2 and 3). High‐altitude group rats were exposed to 4000 m of simulated altitude (4 h/day, 4 weeks) in a chamber (Yantai Binglun Boiler Co. Ltd.; SN: 02–00600‐01; pressure: −0.1 MPa; volume: 0.56 m^3^). All rats exhibited hemoglobin (Hb) concentrations > 180 g/L (mean~200 g/L), confirming successful model establishment [21, 25]. ICH was induced via autologous tail vein blood injection. The target site was the right basal ganglia (relative to the bregma): 1 mm posterior, 3 mm right, and 6 mm deep; 100 μL autologous tail blood was infused [26].

### 2.5 CCL2 Intervention Experiment

To clarify the role of CCL2 in high‐altitude ICH, we conducted the following intervention experiment (Figure S1, Experiment 4):

H‐AAV‐Scramble (control) and H‐AAV‐CCL2‐INT groups (including sham/ICH subgroups): The rats received CCL2‐shRNA AAV (15 μL, BrainVTA, PT‐12112) or scramble control AAV (15 μL, BrainVTA, PT‐0923) via right lateral ventricle injection, followed by 28 days of high‐altitude exposure, and were then subjected to either sham surgery or ICH modeling. H‐sham‐CCL2‐Ab and H‐ICH‐CCL2‐Ab groups: After successful establishment of the high‐altitude model, a CCL2‐neutralizing antibody (10 μg/5 μL, Bioxcell, BE0185) [27] was administered prior to sham surgery or ICH induction. H‐sham‐CCL2 and H‐ICH‐CCL2 groups: Rats in the high‐altitude group were injected with recombinant CCL2 (30 ng/5 μL, Novoprotein, C16A) [28] prior to undergoing sham surgery or ICH induction. After the intervention, we assessed the neurological function and BBB structural/functional changes of the rats.

### 2.6 CCR2 Intervention Experiment

To confirm whether CCL2 disrupts the BBB via the CCL2–CCR2 pathway, we performed CCR2 inhibition experiments (Figure S1, Experiment 5):

H‐sham‐CCR2‐I and H‐ICH‐CCR2‐I groups: H‐group rats received 10 μg (10 μL) of a CCR2 inhibitor (Tocris, RS504393) [29] via basal ganglia injection prior to undergoing sham surgery or ICH modeling H‐sham‐CCL2‐CCR2‐I and H‐ICH‐CCL2‐CCR2‐I groups: H‐group rats received sequential injections of 30 ng (5 μL) recombinant CCL2 and 10 μg (10 μL) CCR2 inhibitor into the basal ganglia prior to undergoing sham surgery or ICH modeling. After inhibition, we assessed the neurological function and BBB structural/functional changes of the rats.

### 2.7 Transcriptomic Experimental Methods

To explore the pathways downstream of the CCL2‐CCR2 axis that mediate BBB damage in high‐altitude ICH, we performed transcriptomics on perihematomal brain tissues (*n* = 6/group) from the H‐sham, H‐ICH, and H‐AAV‐CCL2‐INT‐ICH groups at 1 day post‐ICH (Figure S1, Experiment 6) (Shanghai Majorbio Biopharm Biotechnology Co. Ltd.).

### 2.8 Immunohistochemistry

The rats were anesthetized and transcardially perfused with cold 0.9% saline followed by 4% paraformaldehyde [30]. The brains were processed according to standard protocols, fixed, gradient dehydrated, and sectioned into 30‐μm cryosections. The sections were permeabilized, blocked, and incubated overnight at 4°C with the following primary antibodies: anti‐CCL2 (2H5; 14–7096‐81; Invitrogen), anti‐Iba1 (GTX100042; GeneTex), anti‐ZO‐1 (PB9234; Boster), anti‐CD31 (AF3628; Bio‐Techne), anti‐GFAP (P14136; Bioworld), and anti‐CCR2 (PA5‐23037; Invitrogen). After being washed, the sections were incubated with fluorescent secondary antibodies for 1 h at room temperature and then washed again. Sections were mounted with DAPI‐containing antifade medium (P0131; Beyotime) and imaged via ultrahigh‐resolution confocal microscopy (LSM880; ZEISS).

### 2.9 Western Blot (WB) Analysis

Perihematomal basal ganglia tissues were collected at 1/3/7 days post‐ICH, following perfusion, clot removal, and hippocampal exclusion. Total protein was extracted with Pierce IP Lysis Buffer (87,788; Thermo) supplemented with phosphatase (04906837001; Roche) and protease (04693159001; Roche) inhibitors. 30 μg protein/well was separated by SDS–PAGE and then transferred to PVDF membranes. The membranes were blocked with 5% nonfat milk for 2 h at room temperature and then incubated overnight at 4°C with the following primary antibodies: anti‐CCL2 (PA5‐34505), anti‐ZO‐1 (61–7300), anti‐phospho‐NF‐κB (MA5‐15160), and anti‐NF‐κB (PA5‐16545) (all from Invitrogen). Bands were imaged with Fusion FX Edge Spectra (Vilber Bio Imaging) and quantified using Evolution–Capt Edge software.

### 2.10 Enzyme‐Linked Immunosorbent Assay (ELISA)

To quantify CCL2 in rat peripheral blood and brain tissues, 1 mL of subclavian vein blood was collected in EDTA‐free tubes, followed by centrifugation at room temperature (2 h) and 4°C (1800 × g, 10 min). Brain tissue protein samples were prepared as described for WB. Detection was performed using a rat CCL2 ELISA kit (ELR‐CCL2; RayBiotech, Atlanta, USA) per the manufacturer's instructions.

### 2.11 Brain Water Content

The brain water content was measured at 1/3/7 days post‐ICH. The ipsilateral (hemorrhagic) cerebral hemisphere was dissected for wet weight measurement and then dried at 100°C for 24 h (until constant weight). The brain water content was calculated as follows: [(wet weight−dry weight)/wet weight] × 100%.

### 2.12 Evans Blue (EB) Leakage Assay

BBB permeability was evaluated using an Evans blue tracer (DK0051; Leagene, China), which cannot cross intact BBB. At 1/3/7 days post‐ICH, 2% EB (5 mL/kg) was injected into the tail vein and allowed to circulate for 2 h. The rats were anesthetized and transcardially perfused with 0.9% saline (clear effluent), after which the ipsilateral cerebral hemisphere was harvested. EB content was quantified via a standard curve (normalized to μg/g brain tissue) for assessment of BBB permeability. For semiquantitation, the brain sections were stained with DAPI, and EB leakage was detected via confocal microscopy to confirm BBB damage [29].

### 2.13 Imaging and Histomorphological Analysis

Magnetic resonance (MR) imaging was conducted with a Bruker 7.0 T small‐animal scanner (BioSpec USR 70/20, Paravision 6.0.1, Germany) at the Department of Radiology, Daping Hospital, Army Medical University; the rats were anesthetized with 4% isoflurane, positioned with a four‐channel head coil, and scanned for T1WI/T2WI [31, 32]. For transmission electron microscopy (TEM), euthanized rats were rapidly decapitated to harvest brains; 1 mm^3^ perihematomal tissue (dissected on ice at a fixed distance of 1 mm from the hematoma margin) was fixed in 2.5% glutaraldehyde (4°C) and postfixed with 2% PBS‐dissolved osmium tetroxide, after which TEM images were acquired [33, 34] (Wuhan Servicebio Company, China). Conventional pathological sections were prepared through standard perfusion, fixation and dehydration of brain tissues to further visualize pathological damage after ICH.

### 2.14 Neurofunctional Assessments

Neurological function was evaluated using the modified neurological severity score (mNSS); locomotor activity was assessed by the open field test, and spatial learning and memory were measured by the Morris water maze (MWM) test on day 7 post‐ICH (*n* = 6/group). Detailed methods were described in Figure S9.

### 2.15 Statistical Analysis

The data were analyzed with GraphPad Prism 10, and the results are presented as the means ± SEMs. Two‐way ANOVA (multiple comparisons) was used for intergroup comparisons at different time points (behavioral tests, EB leakage); one‐way ANOVA was used for the WB and ELISA results, and unpaired *t*‐tests were used for two‐group comparisons. Statistical significance was set at *p* < 0.05.

## 3 Results

### 3.1 Olink Proteomics Screening Results

**FIGURE 1:**
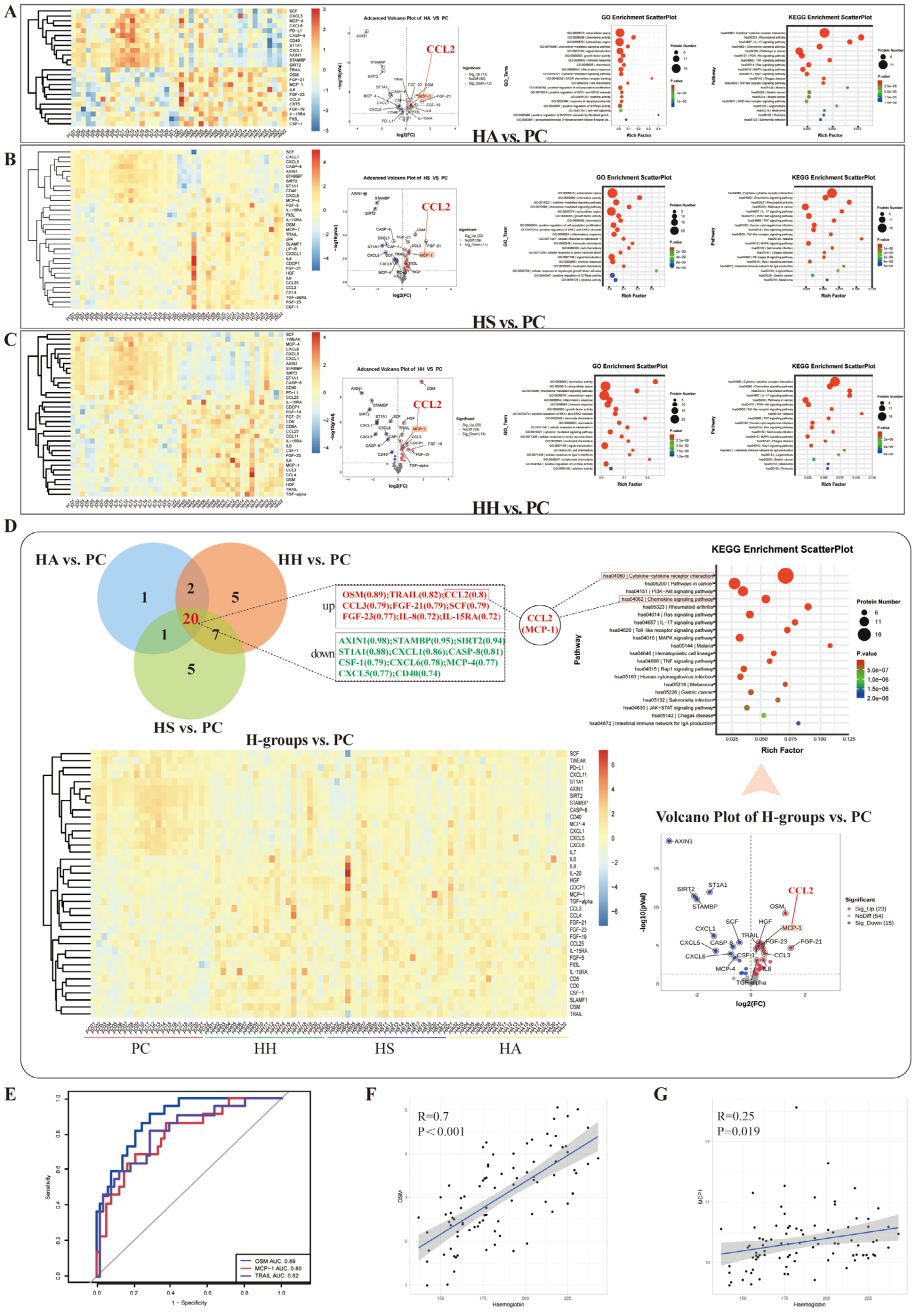
Results of peripheral blood Olink proteomic analysis of different subgroups of high‐altitude immigrants after chronic high‐altitude exposure and plain‐resident controls. Groups: HH (high‐altitude hyperhemoglobinemia); HS (high‐altitude subclinical polycythemia); HA (high‐altitude adaptation); PC‐group (plain control); H‐groups (HH + HS + HA). A–C: Comparisons of the HA/HS/HH groups vs. the PC‐group, respectively. Heatmaps: Differential expression of inflammatory factors across groups. Volcano plots: Significantly altered factors (e.g., CCL2 was prominently upregulated in all plateau groups). GO and KEGG enrichment plots: Annotation of functional pathways associated with differentially expressed factors. D: Integrated analysis of shared DEFs between all plateau subgroups and the PC group. Venn diagram: 10 shared upregulated factors (OSM, CCL2, TRAIL, FGF‐21, CSF‐1, FGF‐23, MCP‐4, IL‐8, CCL3, and IL‐15RA) and 10 downregulated factors (AXIN1, ST1A1, SIRT2, STAMBP, CXCL1, CASP‐8, CXCL6, CD40, SCF, and CXCL5) (all *p* < 0.001). Heatmap/volcano plot (H‐groups vs. PC): Emphasizes CCL2 as a core differential factor. KEGG plot: The top enriched pathway was cytokine–cytokine receptor interaction, with 9 differential chemokines (CCL2 [MCP‐1], IL‐8 [CXCL8], CCL3 [MIP‐1α], CCL4 [MIP‐1β], CCL13 [MCP‐4], CCL25 [TECK], CXCL1, CXCL5, and CXCL6) accounting for the greatest proportion. E: ROC curves for diagnosing plateau‐related phenotypes (H‐groups vs. the PC): OSM (AUC = 0.89) and CCL2 (AUC = 0.80) exhibited favorable diagnostic efficacy. F–G: Pearson correlation analysis: OSM vs. hemoglobin level (*R* = 0.70, *p* < 0.001); CCL2 vs. hemoglobin level (*R* = 0.25, *p* = 0.019).

As shown in Figure 1A–C, all plateau groups (HA/HS/HH) exhibited differential factor expression versus the plain control (PC), with CCL2 showing marked differences across all groups in volcano plots. A Venn diagram (Figure 1D) revealed 10 shared upregulated factors (e.g., CCL2, OSM, and TRAIL) and 10 downregulated factors (all *p* < 0.001). OSM (AUC = 0.89; Figure 1E) strongly correlated with hemoglobin levels (*R* = 0.7; *p* < 0.001; Figure 1F), whereas CCL2 levels (AUC = 0.80) were weakly correlated (R = 0.25; *p* = 0.019; Figure 1G). TRAIL (AUC = 0.82) is a type of protective inflammatory factor [35]. KEGG enrichment (Figure 1D) highlighted cytokine–cytokine receptor interaction. The expression of nine chemokines significantly differed: CCL2, IL‐8, CCL3, CCL4, CCL13, CCL25, CXCL1, CXCL5, and CXCL6. Among them, CCL2, IL8, CCL3, CXCL1, and CXCL5 are inflammatory chemokines. Therefore, we believe that various chemokines may play important roles in the remodeling of the inflammatory network after plateau exposure, and among the numerous chemokines, CCL2 exhibited the most significant difference in expression (AUC = 0.8), making it the most representative inflammatory chemokine. Previous studies have also regarded CCL2 as a marker of plateau injury [13]. Increased CCL2 expression (via HIF‐1α‐mediated pathways) is associated with a variety of high‐altitude diseases, such as HAPH [36], HACE [37], and HAPE. Thus, CCL2 may be involved in multiple pathological processes associated with chronic high‐altitude exposure, including ICH following long‐term high‐altitude exposure.

### 3.2 Verification of CCL2 Expression in Rats Exposed to High Altitude

**FIGURE 2:**
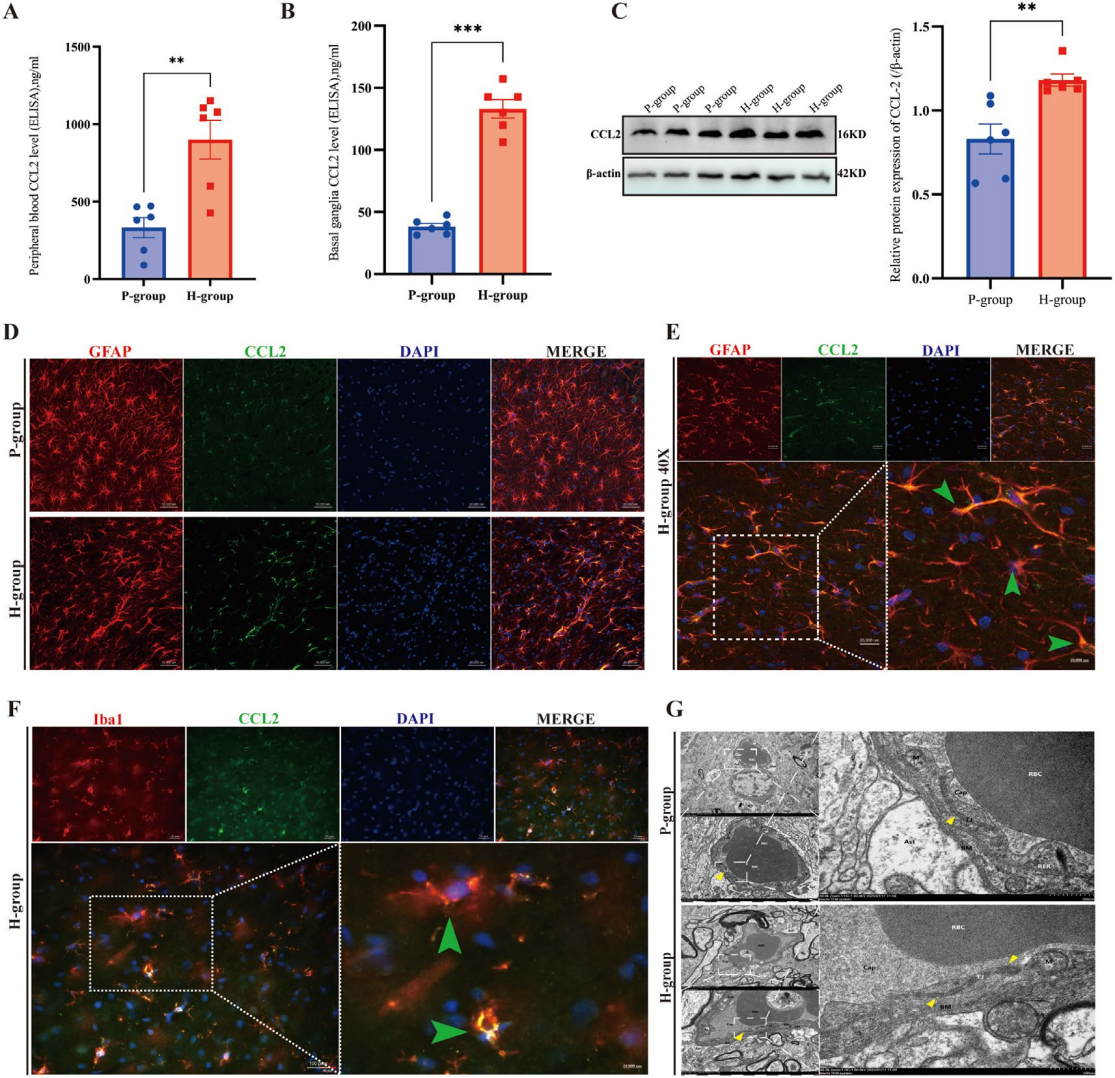
CCL2 expression and blood–brain barrier (BBB) alterations in plain‐altitude (P‐group) vs. high‐altitude (H‐group) rats. Groups: P‐group (rats housed at plain altitude); H‐group (rats exposed to chronic high‐altitude conditions). All experiments: *N* = 6 per group; data are presented as the means ± SEMs. Independent samples *t* tests were used for two‐group parametric comparisons. Significance: **p < 0.01, ***p < 0.001. A. Comparison of CCL2 expression levels in peripheral blood between the P‐group and the H‐group (ELISA) (**p < 0.01). B. Comparison of CCL2 expression levels in basal ganglia brain tissue between the P‐group and the H‐group (ELISA) (***p < 0.001). C. Comparison of CCL2 expression levels in basal ganglia brain tissue between the P‐group and the H‐group (WB) (**p < 0.01). D. Representative images of CCL2 expression in the basal ganglia brain tissue between the P‐group and the H‐group by multiplex fluorescence staining (scale bar = 50 μm): CCL2 (green), GFAP (red), and DAPI (blue). E. CCL2‐astrocyte (GFAP) colabeling (IF; scale bar = 20 μm) in the H‐group: CCL2 (green) predominantly colocalizes with activated GFAP+ astrocytes (red). The green arrows indicate regions in which CCL2 and GFAP are colocalized. F. CCL2‐microglia (Iba1) colocalization in the H‐group (IF; scale bar = 50 μm): A small fraction of CCL2 (green) colocalized with Iba1+ microglia (red). G. BBB ultrastructure (TEM, scale bar = 0.5 μm) in the P‐group and H‐group.

To verify CCL2 expression in a chronic high‐altitude exposure rat model (Figure S1, Experiment 2), we quantified CCL2 in the peripheral blood and basal ganglia of P‐group and H‐group rats. ELISA revealed that CCL2 expression in peripheral blood (*p* < 0.01; *n* = 6; Figure 2A) and the basal ganglia (*p* < 0.001; *n* = 6; Figure 2B) was significantly greater in the H‐group than in the P‐group, as validated by WB analysis of basal ganglia brain proteins (*p* < 0.01; *n* = 6; Figure 2C) and immunofluorescence (Figure 2D). Immunofluorescence staining revealed that CCL2 was colocalized mainly with activated astrocytes (GFAP+; Figure 2E), confirming that astrocytes were the primary source [38]; a small fraction of the cells were colocalized with microglia (Iba1+; Figure 2F). Long‐term high‐altitude exposure caused brain injury: TEM revealed an intact BBB in the P‐group, while mild BBB damage was detected in the H‐group (Figure 2G). Elevated peripheral and intracranial CCL2 levels were correlated and were exacerbated by high‐altitude‐induced BBB damage.

### 3.3 Changes in the Expression of CCL2 and BBB Integrity After ICH in the P‐Group and H‐Group

**FIGURE 3:**
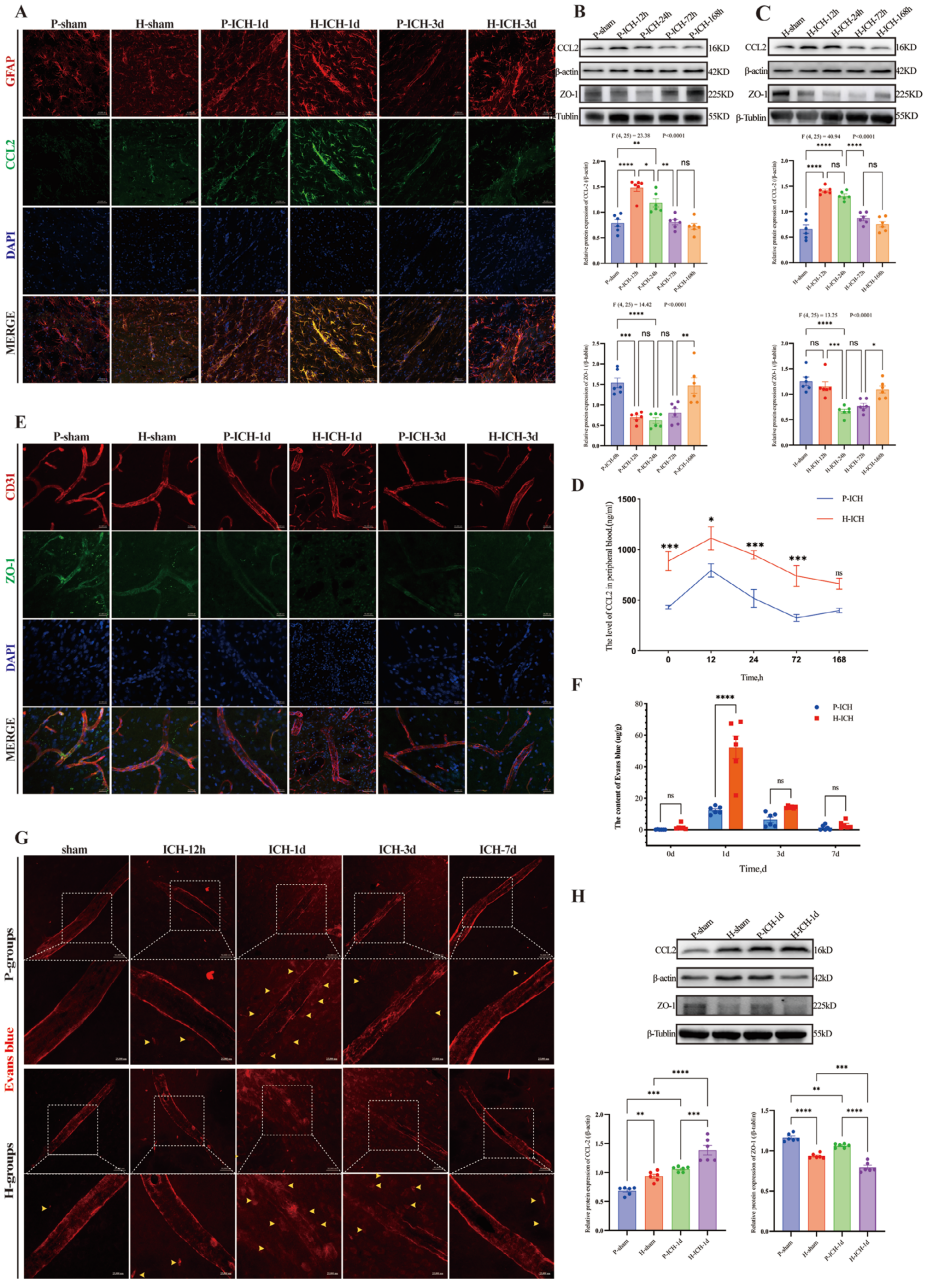
Temporal changes in CCL2 expression and BBB integrity after ICH in P‐group and H‐group rats. A. Representative photomicrographs of CCL2 expression in P‐group and H‐group rats at 1 day and 3 days post‐ICH (scale bar = 50 μm): CCL2 (Green), GFAP (red), and DAPI (blue). B–C. Temporal dynamics of CCL2 and ZO‐1 protein expression (WB): Representative blots (CCL2 + β‐Actin; ZO‐1 + β‐tubulin) and quantitative analyses (normalized to the respective internal controls) for the (B) P‐group and (C) H‐group (*n* = 6; one‐way ANOVA). D. Trends and intergroup comparisons of CCL2 expression levels in peripheral blood from P‐group and H‐group rats at different time points post‐ICH (ELISA) (*n* = 6; two‐way repeated‐measures ANOVA). E. Representative photomicrographs of ZO‐1 expression detected by immunofluorescence staining in P‐group and H‐group rats at 1d and 3d post‐ICH (scale bar = 50 μm): Zo‐1 (green), CD31 (red), and DAPI (blue). F. Trends and intergroup comparisons of quantitative Evans blue (EB) leakage in the perihematomal region of rats from the P‐group and the H‐group at various time points post‐ICH (*n* = 6; two‐way ANOVA). G. Representative qualitative images of EB leakage 1 day post‐ICH in the P‐group and the H‐group (scale bar = 50 μm). H. Comparison of the expression levels of CCL2 and ZO‐1 (normalized to those of β‐Actin or β‐tubulin) between the P‐group and H‐group rats at 1 day post‐ICH (*n* = 6; one‐way ANOVA) by WB. Data are presented as the means ± SEMs; P values: Ns: *P* > 0.05, *p < 0.05, **p < 0.01, ***p < 0.001.

To clarify the link between CCL2 expression and BBB disruption after ICH in P‐group and H‐group rats, we analyzed the temporal dynamics of CCL2, tight junction protein (ZO‐1), and BBB integrity (Figure S1, Experiment 3). In both groups, CCL2 expression peaked at 12 h–1 day post‐ICH in both the perihematomal basal ganglia (Figure 3A–C) and peripheral blood (Figure 3D). Concordantly, ZO‐1 expression was lowest at 1 day post‐ICH (Figure 3B,C,E), and Evans blue extravasation also peaked at 1 day post‐ICH (Figure 3F,G). These temporally aligned trends suggest a potential association between CCL2 dynamics and BBB disruption. At 1 day post‐ICH (the time point of maximal CCL2 and BBB disruption), WB (Figure 3H) revealed that the H‐group had significantly higher CCL2 levels (*p* < 0.001; *n* = 6) but lower ZO‐1 levels (*p* < 0.001; *n* = 6) than the P‐group did. Collectively, these findings indicate that elevated CCL2 expression (peaking at 12 h–1 day post‐ICH) may exacerbate post‐ICH BBB disruption [39, 40] by downregulating ZO‐1 expression and that high‐altitude exposure likely amplifies this pathogenic process.

### 3.4 Comparison of Cerebral Edema and Neurobehavioral Changes at Different Time Points After ICH in P‐Group and H‐Group Rats

Brain water content assays revealed that compared with the P‐group, the H‐group developed significantly more severe edema as early as 1 day post‐ICH (*p* < 0.01; *n* = 6), with this severity persisting through 7 days post‐ICH (*p* < 0.01; *n* = 6) (Figure S2A). Cerebral edema is typically correlated with neurological deficits; thus, we assessed the neurobehavioral outcomes and observed that compared with the P‐group, the H‐group showed consistently greater mNSS (reflecting worse deficits; *p* < 0.001; *n* = 6; Figure S2B), reduced 5 min total travel distance in the open field test (indicating impaired motor function; *p* < 0.001; *n* = 6; Figure S2C,D), and worse 7 days post‐ICH MWM test performance (longer escape latency during training [*p* < 0.05; *n* = 6], fewer platform crossings, and less target quadrant time in the probe trial [both *p* < 0.01; *n* = 6]); the swimming speed was comparable between the groups (*p* > 0.05), confirming the learning and memory deficits (Figure S2E,F). The morphological basis of edema was characterized, and the multidimensional assessments consistently revealed worse pathology in the H‐group. TEM revealed more severe BBB disruption (Figure S2G) and basement membrane and organelle damage at 1 day post‐ICH (with edema exacerbated by 3 days), and MRI demonstrated more prominent edema at 3 days post‐ICH (the edema peak) (Figure S2H). Pathological sections revealed delayed hematoma absorption in the H‐group vs early absorption in the P‐group at 3 days post‐ICH (Figure S2I). Collectively, these findings indicate that the high‐altitude group rats exhibited more severe, persistent cerebral edema and worse neurobehavioral deficits post‐ICH; these outcomes are likely linked to elevated CCL2 levels in the H‐group, a relationship that merits further mechanistic investigation.

### 3.5 CCL2 Interventions to Explore the Relationships Between CCL2 Expression and Blood–Brain Barrier Disruption, Cerebral Edema and Neurobehavioral Changes

**FIGURE 4:**
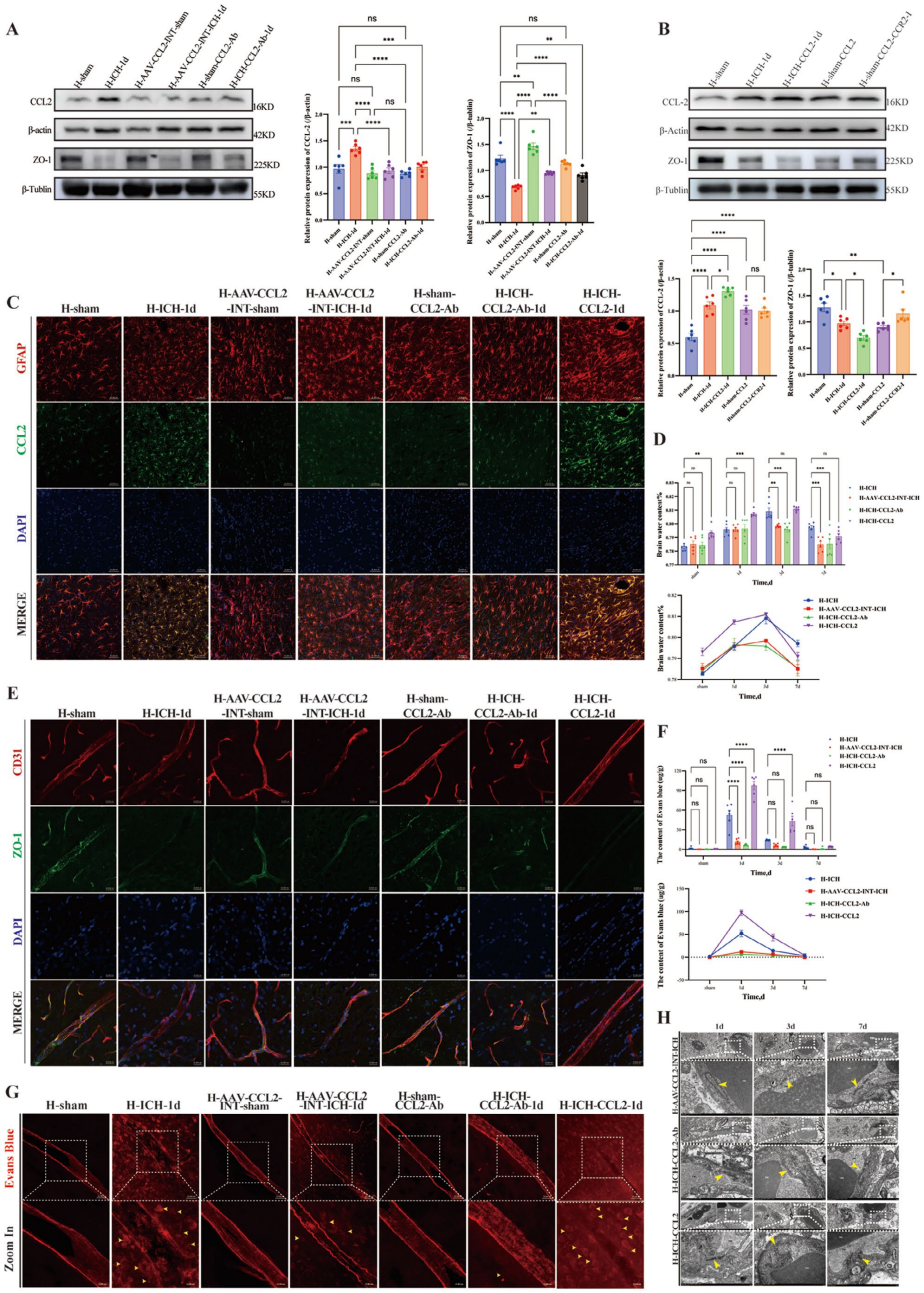
Effects of CCL2 intervention on BBB integrity and cerebral edema in high‐altitude rats after ICH. Groups: H‐sham, H‐ICH‐1d; AAV‐mediated CCL2 knockdown: H‐AAV‐CCL2‐INT‐sham, H‐AAV‐CCL2‐INT‐ICH; CCL2‐neutralizing antibody treatment: H‐sham‐CCL2‐Ab, H‐ICH‐CCL2Ab; recombinant CCL2 supplementation: H‐sham‐CCL2, H‐ICH‐CCL2. A. WB analysis of CCL2 and ZO‐1 expression in the CCL2 knockdown groups: Blots (left): CCL2 + β‐Actin; ZO‐1 + β‐tubulin for the groups in this panel. Quantification (right bar graphs): Relative protein levels (*n* = 6; one‐way ANOVA). B. WB analysis of CCL2 and ZO‐1 expression in the groups treated with recombinant CCL2: Blots: CCL2 + β‐Actin; ZO‐1 + β‐tubulin for the groups in this panel. Quantification: Relative protein levels (*n* = 6; one‐way ANOVA). C. Representative photomicrographs of CCL2 expression detected by immunofluorescence staining in each group at 1 day post‐ICH following CCL2 intervention (scale bar = 50 μm): CCL2 (Green), GFAP (red), and DAPI (blue). D. Bar graph and line graph of the results of the statistical analysis of the brain water content in each group after CCL2 intervention (*n* = 6; two‐way ANOVA). E. Representative photomicrographs of ZO‐1 expression detected by immunofluorescence staining in each group at 1 day post‐ICH following CCL2 intervention (scale bar = 50 μm): Zo‐1 (green), CD31 (red), and DAPI (blue). F. Qualitative analysis of the EB leakage assay results at 1 day, 3 days, and 7 days post‐ICH after the three interventions (*n* = 6; two‐way ANOVA). G. Representative qualitative images of EB leakage in each group at 1 day post‐ICH following CCL2 intervention (scale bar = 50 μm). H. Representative images of BBB ultrastructure (TEM; scale bar = 0.5 μm) in each group at 1 day, 3 days, and 7 days post‐ICH following CCL2 intervention (yellow arrows indicate key BBB components). Data are presented as the means ± SEMs; P values: Ns: *P* > 0.05, *p < 0.05, **p < 0.01, ***p < 0.001.

On the basis of the concurrent peak in CCL2 expression and the downregulation of ZO‐1 expression at 1 day post‐ICH, we targeted CCL2 in H‐group rats, namely, those in which CCL2 was knocked down (H‐AAV‐CCL2‐INT‐ICH and H‐ICH‐CCL2‐Ab) or upregulated (H‐ICH‐CCL2) (Figure S1, Experiment 4). AAV was accurately expressed in the basal ganglia surrounding the lateral ventricles following injection (Figure S3A). TEM revealed that the integrity of the BBB was significantly better in the H‐AAV‐CCL2‐INT‐sham group than in the H‐sham and H‐sham‐CCL2‐Ab groups (Figure S3B). Compared with the H‐sham subgroup, all the sham subgroups exhibited significant differences in CCL2 expression (*p* < 0.01; Figure S3C). Compared with H‐ICH, H‐AAV‐CCL2‐INT‐ICH and H‐ICH‐CCL2‐Ab reduced CCL2 expression (*p* < 0.001, *n* = 6) and elevated ZO‐1 expression (*p* < 0.01, *n* = 6) (Figure 4A,C,E) whereas H‐ICH‐CCL2 increased CCL2 expression (*p* < 0.05, *n* = 6) and decreased ZO‐1 expression (*p* < 0.05, *n* = 6) (Figure 4B,C,E). Pathologically, CCL2 inhibition alleviated cerebral edema at 3 days (*p* < 0.01; *n* = 6) (Figure 4D) and vascular leakage at 1 day (*p* < 0.001; *n* = 6) (Figure 4F,G), with TEM confirming relatively intact tight junctions at 1 and 3 days (Figure 4H). H‐ICH‐CCL2 treatment resulted in severe BBB disruption (tight junction/basement membrane dissolution) and leakage (*p* < 0.001; *n* = 6) at 1 day but no significant difference in edema at 3 and 7 days (*p* > 0.05) (Figure 4F–H).

Behaviorally, the CCL2‐inhibited groups had lower 1–7‐day mNSS (*p* < 0.01; *n* = 6) (Figure S4A), longer 5 min open‐field travel distances (*p* < 0.001; *n* = 6) (Figure S4B), and better performance on the 7‐day MWM test (shorter escape latency, longer target quadrant time, and more platform crossings; all *p* < 0.01; *n* = 6) (Figure S4C). H‐ICH‐CCL2 mice had a greater 0.5‐day mNSS (*p* < 0.05; *n* = 6) and a longer 7‐day training latency (*p* < 0.01; *n* = 6) (Figure S4A,B).

Collectively, these findings suggest that CCL2 upregulation exacerbates BBB disruption after high‐altitude ICH, whereas CCL2 blockade mitigates BBB injury, cerebral edema, and neurological deficits (motor/learning/memory), suggesting that CCL2 is a key pathogenic factor in high‐altitude ICH.

### 3.6 Blockade of CCR2 to Investigate Changes in the Blood–Brain Barrier (BBB), Cerebral Edema, and Neurobehavior

**FIGURE 5:**
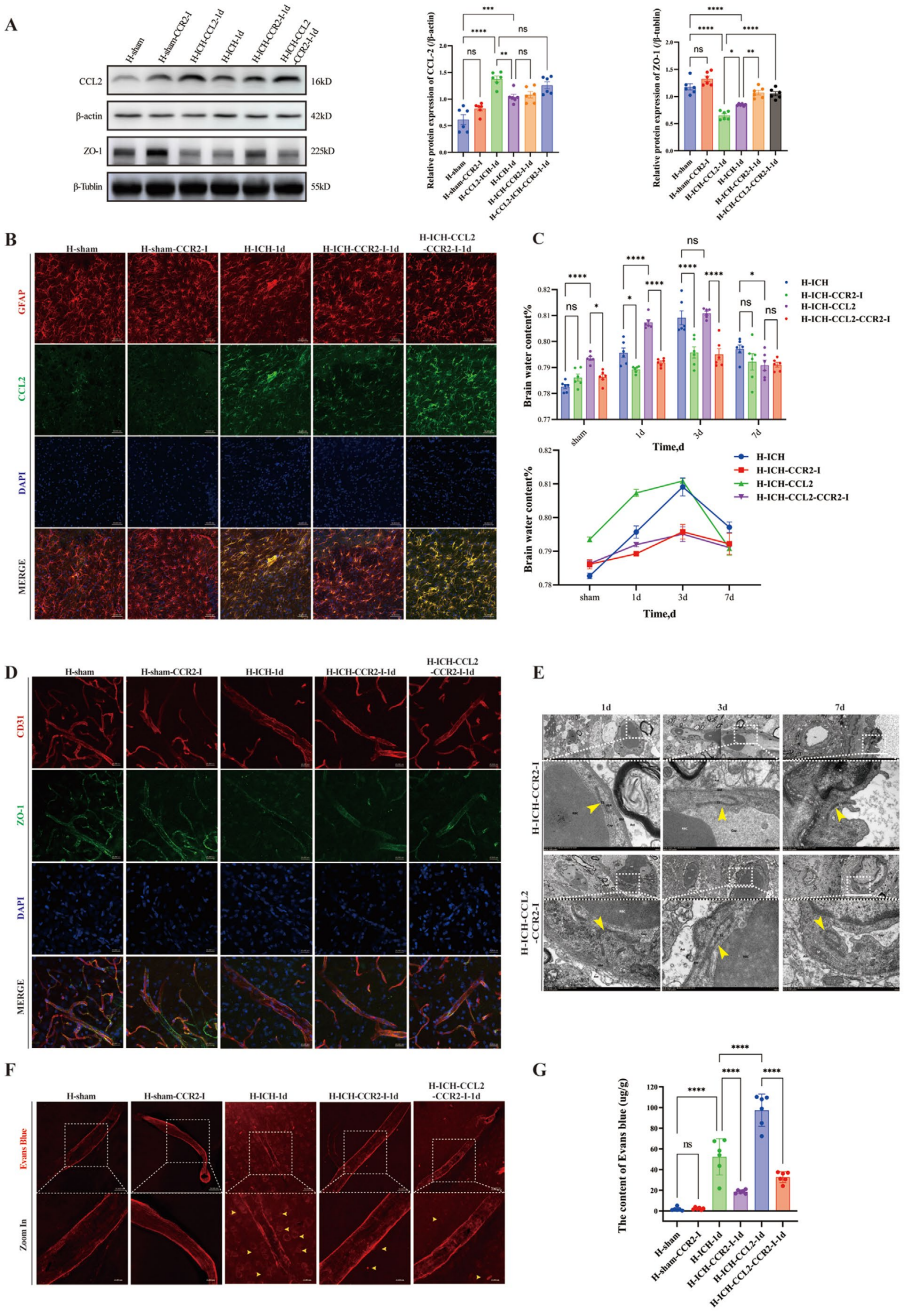
Effects of CCR2 blockade on BBB integrity and cerebral edema in high‐altitude rats after ICH. Groups: H‐sham, H‐ICH‐1d; CCR2 blockade (CCR2‐I): H‐sham‐CCR2‐I, H‐ICH‐CCR2‐I, H‐ICH‐CCL2‐CCR2‐I. A. Representative blots and statistical analysis of CCL2 and ZO‐1 protein expression levels (normalized to β‐Actin or β‐tubulin) in perihematomal brain tissue from each group 1 day post‐ICH after CCR2 blockade (WB, *n* = 6; one‐way ANOVA). B. Representative photomicrographs of CCL2 expression detected by immunofluorescence staining in each group at 1 day post‐ICH following CCR2 blockade (scale bar = 50 μm): CCL2 (Green), GFAP (red), and DAPI (blue). C. Bar graph and line graph showing the results of the statistical analysis of the brain water content for each group following CCR2 blockade (*n* = 6; two‐way ANOVA). D. Representative photomicrographs of ZO‐1 expression detected by immunofluorescence staining in each group at 1 day post‐ICH following CCR2 blockade (scale bar = 50 μm): Zo‐1 (green), CD31 (red), and DAPI (blue). E. Representative images of BBB ultrastructure (TEM; scale bar = 0.5 μm) in each group at 1 day, 3 days, and 7 days post‐ICH following CCR2 blockade (yellow arrows indicate key BBB components). F. Representative qualitative images of EB leakage in each group at 1 day post‐ICH following CCR2 blockade (scale bar = 50 μm; yellow arrows = leakage regions). G. Quantitative statistical analysis of EB leakage in each group after CCR2 blockade (*n* = 6; one‐way ANOVA). Data are presented as the means ± SEMs; P values: Ns: *P* > 0.05, *p < 0.05, **p < 0.01, ***p < 0.001.

To clarify the mechanism through which CCL2 affects the BBB, we inhibited the expression of CCR2 (CCL2s cognate receptor) and characterized its downstream changes (Figure S1, Experiment 5). WB (Figure 5A) and immunofluorescence (Figure 5B) confirmed that CCR2 blockade (H‐sham‐CCR2‐I, H‐ICH‐CCR2‐I, H‐ICH‐CCL2‐CCR2‐I) did not alter CCL2 expression compared with that in the respective controls (H‐sham, H‐ICH, H‐ICH‐CCL2) (*p* > 0.05; *n* = 6) but significantly upregulated ZO‐1 expression (H‐ICH‐CCR2‐I vs. H‐ICH: *p* < 0.01; H‐ICH‐CCL2‐CCR2‐I vs. H‐ICH‐CCL2: *p* < 0.001; *n* = 6; Figure 5A–D). TEM (Figure 5E) revealed preserved BBB integrity (intact basement membranes/tight junctions and alleviated mitochondrial edema) at 3 days post‐ICH. The brain water content (3 days post‐ICH) and EB leakage (1 day post‐ICH) were lower in the CCR2‐blocked groups than in the respective control groups (*p* < 0.001; *n* = 6; Figure S5C,F,G; H‐ICH‐CCL2 leakage: Figure 4F,G). Behaviorally, the CCR2‐blocked groups had improved mNSS (Figure S5A), longer 1–3 days open‐field 5 min travel distances (*p* < 0.001; *n* = 6; Figure S5B), and better performance on the 7 days MWM test (Figure S5C). After CCR2 blockade, the expression of CCR2 significantly decreased (*p* < 0.001; *n* = 6; Figure S6A,B).

To exclude the potential confounding effects of solvents (0.1% DMSO for the CCR2 inhibitor, 5% BSA for the CCL2‐neutralizing antibody, and 0.9% saline for recombinant CCL2), sham surgery, and control AAV‐scramble on our findings, we performed dedicated WB experiments, which demonstrated that these solvents had no significant effect on the expression of CCL2 or ZO‐1 (*p* > 0.05, *n* = 6) (Figure S7).

Collectively, these findings suggest that CCL2 drives BBB disruption, cerebral edema, and neurological deficits primarily via the CCL2–CCR2 pathway: CCR2 inhibition upregulates ZO‐1, thereby preserving BBB integrity, reducing permeability/edema, and ameliorating neurobehavioral dysfunction.

### 3.7 Transcriptomics Reveals the Pathological Mechanisms of CCL2 in High‐Altitude ICH and the Effects of CCL2 Knockdown

**FIGURE 6:**
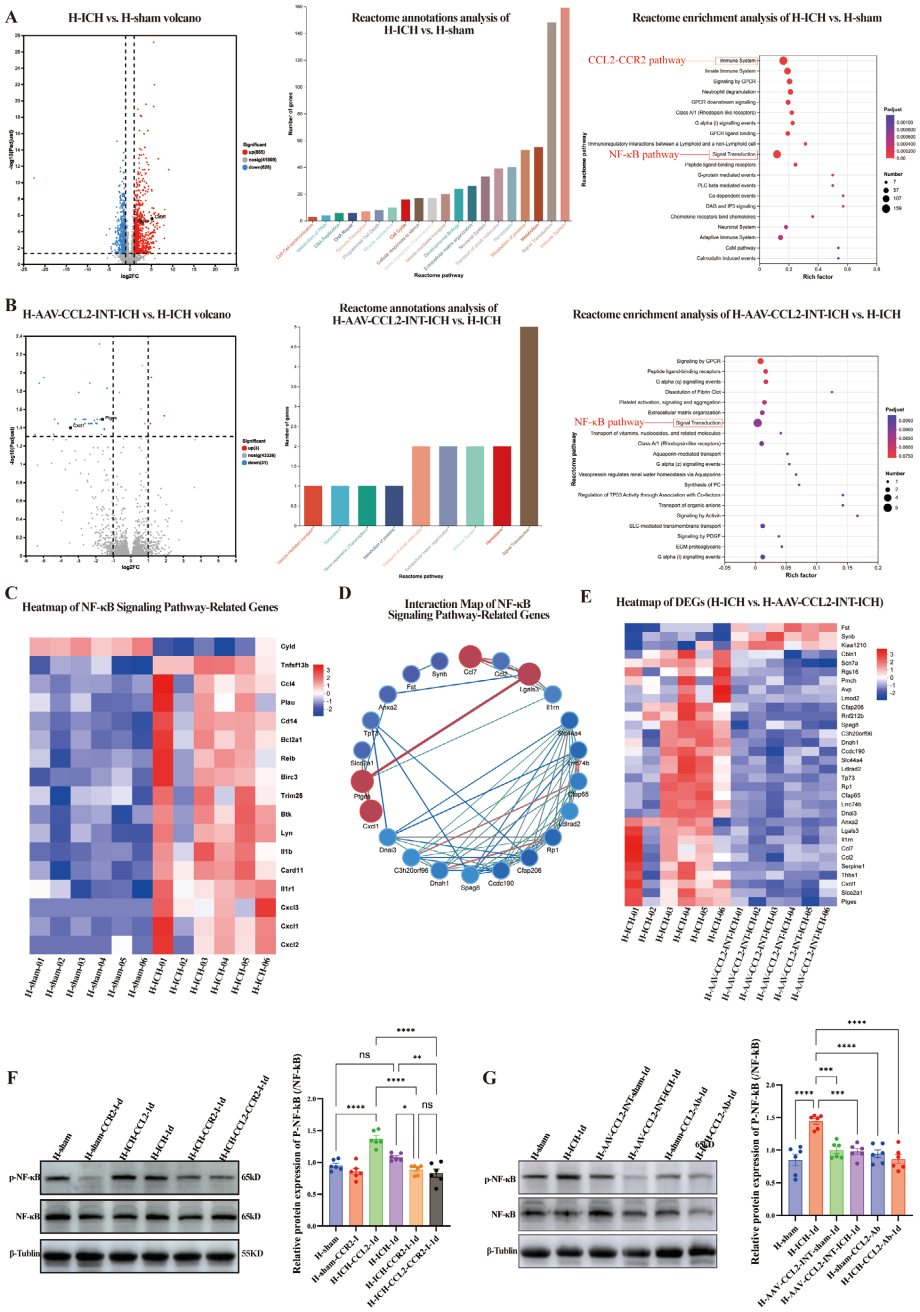
Transcriptomic study of the downstream signaling of the CCL2‐CCR2 pathway. Groups: H‐sham, H‐ICH, and H‐AAV‐CCL2‐INT‐ICH (AAV‐mediated CCL2 knockdown). A. Volcano plot, Reactome functional annotation analysis and Reactome pathway enrichment analysis of the DEGs between the H‐ICH and H‐sham groups. B. Volcano plot, Reactome functional annotation analysis and Reactome pathway enrichment analysis of the DEGs between the H‐AAV‐CCL2‐INT‐ICH and H‐ICH groups. C. Heatmap of DEGs related to the NF‐κB signaling pathway between the H‐ICH and H‐sham groups. D. Interaction map of DEGs related to the NF‐κB signaling pathway between the H‐ICH and H‐AAV‐CCL2‐INT‐ICH groups. E. Heatmap of the DEGs between the H‐ICH and H‐AAV‐CCL2‐INT‐ICH groups. F. WB images and statistical analysis of the relative gray values of P‐NF‐κB/NF‐κB in each group in the CCR2 blockade experiment (*n* = 6; one‐way ANOVA). G. WB images and statistical analysis of the relative gray values of P‐NF‐κB/NF‐κB in each group in the CCL2 intervention experiment (*n* = 6; one‐way ANOVA). Data are presented as the means ± SEMs; P values: Ns: *P* > 0.05, *p < 0.05, **p < 0.01, ***p < 0.001.

To clarify the pathogenic mechanism of CCL2 in high‐altitude ICH, we performed a transcriptomic analysis (Figure 6) of three groups (*n* = 6/group): H‐sham (control), H‐ICH (ICH), and H‐AAV‐CCL2‐INT‐ICH (ICH + CCL2 knockdown). Comparisons revealed H‐ICH vs. H‐sham DEGs were enriched in immune/inflammatory (NF‐κB, IL‐17, and chemokine signaling), signal transduction, neurovascular function, and metabolic pathways (Figure 6A,C).

Compared with H‐ICH, H‐AAV‐CCL2‐INT‐ICH resulted in downregulation of the expression of 29 genes, many of which are involved in NF‐κB activation and its downstream inflammatory targets (e.g., *Cxcl1*, *Ccl7*, *Ptges*, and *Lgals3*), and the upregulation of 3 genes (including *Fst*, which has been reported to have anti‐inflammatory/anti‐fibrotic activity; Figure 6B,D). Mechanistically, elevated CCL2 in H‐ICH binds CCR2 to activate NF‐κB, triggering immune infiltration (*Icam1*) [41], tight junction degradation (*Mmp2*), and neuronal apoptosis [42]; in addition, CCL2 knockdown blocks this axis (Figure 6B,D,E). WB validation confirmed that the p‐NF‐κB/NF‐κB ratio was reduced in the CCR2‐blocked and CCL2‐intervened groups (H‐ICH‐CCR2‐I vs. H‐ICH: *p* < 0.05; H‐AAV‐CCL2‐INT‐ICH vs. H‐ICH: *p* < 0.001; Figure 6F,G), which is consistent with the role of NF‐κB in BBB disruption (immune infiltration, tight junction degradation, inflammation cascade) [43]. Comparative p‐NF‐κB/NF‐κB data for the H‐group vs. P‐group are provided in Figure S8.

## 4 Discussion

Chronic hypoxia in high‐altitude migrants drives baseline pathologies (e.g., proinflammatory factor overexpression) that induce vascular remodeling [15] and coagulopathy [44, 45], contributing to high ICH morbidity and mortality. Notably, even with optimal management (e.g., timely hematoma evacuation, oxygen supplementation, and plain‐area transfer), secondary ICH injury persists, and long‐term neurological deficits (motor dysfunction and cognitive dysfunction) remain. Studies have suggested that humans cannot achieve complete adaptation to long‐term high‐altitude exposure [46]; instead, these conditions can lead to cumulative damage [47]. Our Olink proteomics data directly support this finding: the expression of proinflammatory factors (centered on CCL2) was elevated in plasma from high‐altitude migrants, establishing the pathological basis prerequisite for investigating high‐altitude ICH mechanisms. High‐altitude environmental injury to the brain ranges from disruption of angiogenesis to disruption of the blood–brain barrier (BBB), the induction of immune cell activation and, ultimately, the induction of neuronal apoptosis, and these interactive mechanisms are being increasingly elucidated [15]. These findings highlight the need to investigate high‐altitude ICH from the perspective of vascular–immune–neural network remodeling [48], as well as explore the increasing interest in post‐ICH anti‐inflammatory interventions (e.g., low‐dose dexamethasone [49, 50]) and the targeting of neuroinflammation [51]. Using Olink proteomics, we identified CCL2 as a key factor–with targeted, innovative screening–and excluded the correlation of CCL2 with high‐altitude polycythemia (avoiding confounders) to establish an environmental sensitivity–pathological relevance evidence chain. Mechanistically, CCL2 disrupts the BBB (as evidenced by EB leakage and TEM): binding to CCR2 induces endothelial cytoskeletal rearrangement and ZO‐1 degradation [29, 38]. Additionally, CCL2 recruits activated microglia [52] and monocytes, amplifying inflammation via the activity of IL‐1β/TNF‐α. The more severe secondary injury following ICH in the H‐group arises from the sequential synergistic effects of two processes mediated by chronically elevated baseline CCL2 in high‐altitude environments: inducing basal BBB disruption (thereby lowering the brain injury threshold) and amplifying post‐ICH inflammatory storms, with the latter playing a more critical role. The expression levels of ZO‐1 in the H‐sham group were significantly lower than those in the P‐group (Figure 3H), and mild structural damage to the BBB was observed by TEM (Figure 2G). In contrast, in the H‐AAV‐CCL2‐INT‐sham group, with long‐term suppression of baseline CCL2 expression, CCL2 expression was further reduced, ZO‐1 expression was restored, and the BBB structure was more intact (Figure S3B,C; Figure 4A). These findings confirm that baseline CCL2 serves as a persistent driver of basal BBB impairment. This impairment renders the BBB vulnerable, laying a structural foundation for exacerbated injury after ICH. The superposition of baseline CCL2 levels and acutely elevated CCL2 levels post‐ICH amplifies inflammatory storms via the CCL2‐CCR2‐NF‐κB pathway, which constitutes the core mechanism underlying aggravated injury. The sequential synergy of these two processes forms the unique injury pattern of high‐altitude ICH. Previous studies have shown that blocking the CCL2–CCR2–NF‐κB pathway alleviates neuroinflammation in subarachnoid hemorrhage (SAH) [53]. This study advances the understanding of high‐altitude ICH in three ways: First, it integrates inflammatory factor biology with BBB regulation in the context of high‐altitude ICH to elucidate the underlying mechanism. Second, it extends the role of CCL2 in high‐altitude diseases (previously linked only to cardiovascular conditions [54, 55]), validating it as an environment–CNS injury bridge molecule (at the human/animal level) and refining the pathological network of CCL2 in high‐altitude disorders. Third, the results of combined interventions (AAV‐mediated CCL2 knockdown, neutralizing antibody blockade, and recombinant CCL2 supplementation) support a causal role for CCL2 in high‐altitude ICH pathogenesis and provide evidence to move beyond consideration of CCL2 solely as a biomarker and recognize it as a potential therapeutic target. We acknowledge the following limitations: flow cytometry/immune cell sorting was not performed, precluding full characterization of inflammatory cell (neutrophil/T‐cell [56]) activation within the vascular–immune–neural network (e.g., CCL2‐mediated regulatory T‐cell recruitment [57, 58]). Future research will use scRNA‐seq to analyze immune cell/endothelial cell heterogeneity in high‐altitude ICH and explore the effects of high‐altitude hypoxia on cell fate and pathological phenotype remodeling.

## 5 Conclusion

Using high‐altitude migrants and rat models, we demonstrated that CCL2 is a key inflammatory factor exacerbating high‐altitude ICH injury: long‐term high‐altitude exposure increases baseline CCL2 levels (inducing mild brain damage), and ICH triggers a sharp increase in CCL2 levels. Through the CCL2–CCR2–NF‐κB axis, CCL2 drives inflammatory storm amplification, BBB disruption, and neurological deficits, worsening brain injury. Inhibiting CCL2 or its pathway mitigates high‐altitude post‐ICH injury, offering a novel target for the clinical prevention and treatment of high‐altitude ICH.

## Author Contributions

Rongsu Huang: conceptualization, data curation, formal analysis, investigation, methodology, validation, visualization, writing – original draft; Ling Gao: Data curation, formal analysis, methodology, resources, software, validation, visualization, writing – review and editing; Yalan Dai: data curation, formal analysis, investigation, methodology, validation, visualization, writing – original draft; Shangshi Li: conceptualization, project administration, resources; Jiancai Guru: conceptualization, project administration, resources; Mingxi Li: investigation, methodology; Yù‐Jié Chen: investigation, methodology; Bo Wu: investigation, methodology; Xufang Ru: investigation, methodology, project administration; Qing Gong: investigation, methodology: Jun Tang: investigation, methodology; Gang Zhu: conceptualization, funding acquisition, project administration, resources, supervision, writing – review and editing; Yujie Chen: conceptualization, data curation, funding acquisition, investigation, methodology, project administration, resources, supervision, validation, visualization, writing – review and editing.

## Funding

This work was supported by the National Natural Science Foundation of China (81873754 to Gang Zhu and 82371333 to Yujie Chen), the Natural Science Foundation of Chongqing (Grant No. CSTB2025NSCQ‐LZX0044 to Yujie Chen), the Chongqing Municipal Health Commission (Grant No. YXGD202451 to Yujie Chen), and the Chongqing Talents Project (425Z2P113 to Gang Zhu). The funders played no role in the study design; in the collection, analysis, or interpretation of the data; in the writing of the report; or in the decision to submit the article for publication.

## Ethics Statement

The human sample collection was approved by the Ethics Committee of the General Hospital of Tibet Military Command (Approval No. 24‐Ke‐007‐01) and was performed in accordance with the Declaration of Helsinki. Informed consent to participate in the study was obtained from the participants before collection. All experiments in this study were approved by the Laboratory Animal Welfare and Ethics Committee of the Army Medical University (Approval No. AMUWEC20232224) and reported in accordance with the ARRIVE 2.0 guidelines.

## Consent

Informed consent for publication regarding the human samples was obtained from participants, along with informed consent to participate.

## Conflicts of Interest

The authors declare no conflicts of interest.

## Supporting information


**Figure S1:** Schematic diagram of the experimental design and research methods.
P‐group: plain group; H‐group: high‐altitude group; ICH: intracerebral hemorrhage; WB: Western blot; ELISA: enzyme‐linked immunosorbent assay; IF: frozen section immunofluorescence; TEM: transmission electron microscopy; MWM: Morris water maze test; BWC: brain water content; mNSS: modified neurological severity score; EB: Evans blue; CCL2‐INT‐AAV: adeno‐associated virus (AAV) vector expressing CCL2‐targeted shRNA; r‐CCL2: recombinant CCL2 protein; CCR2‐I: CCR2‐specific inhibitor; CCL2‐Ab: CCL2 neutralizing antibody; BSA: bovine serum albumin; DMSO: dimethyl sulfoxide.
**Figure S2:** Cerebral edema and neurobehavioral changes post‐ICH in P‐group vs. H‐group rats.
A. Statistical analysis of brain water content in P‐group and H‐group rats at 1d, 3d, and 7d post‐ICH (*n* = 6; two‐way ANOVA (Bonferroni post hoc)). B. Trends and intergroup comparisons of modified Neurological Severity Score (mNSS) between the P‐group and the H‐group rats at various time points post‐ICH (*n* = 6; repeated‐measures two‐way ANOVA with Bonferroni post hoc test). C. Changes in 5‐min locomotor distance and intergroup comparisons in the open field test of rats in the P‐group and H‐group at 1d, 3d, and 7d post‐ICH (*n* = 6; repeated‐measures two‐way ANOVA). D. Representative images of the open field test between the P‐group and the H‐group rats at 1d, 3d, and 7d post‐ICH. E. Morris water maze (MWM) trajectories: Upper panels (Learning phase) and lower panels (Memory phase) show paths of P‐sham, P‐ICH‐7d, H‐sham, H‐ICH‐7d rats. F. Statistical analysis of MWM test results in P‐group and H‐group rats on day 7 post‐ICH (platform crossovers, escape latency, target quadrant time, average speed) (*n* = 6, one‐way ANOVA); G. Representative images of BBB ultrastructure (TEM, scale bar = 0.5 μm) in the P‐group and H‐group rats at 1d, 3d, 7d post‐ICH; yellow arrows mark BBB structure. H. Representative MRI (T2) images of P‐group and H‐group rats at 1d and 3d post‐ICH. **I**. Representative images of pathological sections from P‐group and H‐group rats at 1d and 3d post‐ICH. Data are presented as mean ± SEM; P values: ns: *p* > 0.05, *p < 0.05, **p < 0.01, ***p < 0.001.
**Figure S3:** Effects of CCL2 intervention on CCL2 expression and blood–brain barrier ultrastructure in sham‐operated rats.
A. The expression of the virus in the H‐AAV‐CCL2‐INT group after intracerebroventricular injection of AAV virus and subsequent intracerebral hemorrhage (ICH) modeling is shown in the figure, indicated by the red fluorescence inherent to the virus. B. TEM images of BBB ultrastructure in sham‐operated rats from each group: H‐sham, H‐CCL2 Ab‐sham, and H‐AAV‐CCL2‐INT‐sham. Yellow arrows indicate tight junction structures (T), showing differences in the integrity of interendothelial tight junctions across intervention groups. C. WB analysis and quantitative comparison of relative CCL2 protein expression (normalized to β‐Actin) in brain tissues of sham‐operated rats from each group. The highest CCL2 expression was observed in the H‐sham‐CCL2 group, while the lowest expression was detected in the H‐AAV‐CCL2‐INT‐sham group, and expression was significantly downregulated in the H‐sham‐CCL2 Ab group. Data are presented as mean ± SEM; P values: ns: *p* > 0.05, *p < 0.05, **p < 0.01, ***p < 0.001.
**Figure S4:** Neurobehavioral changes in each group after CCL2 intervention.
A. Bar graph and line graph of the results of the statistical analysis of the mNSS in each group after CCL2 intervention (*n* = 6; repeated‐measures two‐way ANOVA). B. Activity trajectory plots of the 5‐min open field test in each group after CCL2 intervention. Bar graph and line graph of the statistical analysis of the 5‐min total distance traveled in the open field test in each group after CCL2 intervention (*n* = 6; two‐way ANOVA). C. Trajectory plots and statistical analysis of the results of the Morris water maze test for each group after CCL2 intervention (*n* = 6; one‐way ANOVA). Data are presented as mean ± SEM; *p* values: ns: *p* > 0.05, *p < 0.05, **p < 0.01, ***p < 0.001.
**Figure S5:** Changes in neurobehavior after blocking CCR2.
A. Bar graph and line graph showing the results of the statistical analysis of the mNSS for each group following CCR2 blockade (*n* = 6; repeated‐measures two‐way ANOVA). B. Five‐minute activity trajectory plots from the open field test for each group following CCR2 blockade. Bar graph and line graph showing the results of the statistical analysis of the 5‐min total distance traveled in the open field test for each group following CCR2 blockade (*n* = 6; two‐way ANOVA). C. Morris water maze test results. Representative trajectory plots of the training phase and probe trial of the MWM test in each group on day 7 after ICH following CCR2 blockade are shown. Statistical analysis of escape latency, number of platform crossings, time spent in the target quadrant, and average speed (*n* = 6; one‐way ANOVA); Data are presented as mean ± SEM; *p* values: ns: *p* > 0.05, *p < 0.05, **p < 0.01, ***p < 0.001.
**Figure S6:** Changes in CCR2 expression following CCR2 blockade.
A. Representative photomicrographs of CCR2 expression detected by immunofluorescence staining in each group at 1 day post‐ICH following CCR2 blockade (scale bar = 50 μm): CCR2 (green), CD31 (red), DAPI (blue). B. Representative blots and statistical analysis of CCR2 protein expression levels (normalized to β‐Tubulin) in perihematomal brain tissue from each group 1 day post‐ICH after CCR2 blockade (WB, *n* = 6; one‐way ANOVA). Data are presented as mean ± SEM; *p* values: ns: *p* > 0.05, *p < 0.05, **p < 0.01, ***p < 0.001.
**Figure S7:** Effects of different solvents on CCL2 and ZO‐1 expression.
WB analysis of CCL2 and ZO‐1 in different solvents groups rats: Blots: CCL2 + β‐actin; ZO‐1 + β‐Tubulin for groups in this panel. Quantification: Relative protein levels (*n* = 6; one‐way ANOVA). Data are presented as mean ± SEM; *p* values: ns: *p* > 0.05, *p < 0.05, **p < 0.01, ***p < 0.001.
**Figure S8:** Changes in p‐NF‐κB/NF‐κB expression between the P‐group and H‐group on day 1 post‐ICH.
WB images and statistical analysis of the relative gray values of P‐NF‐κB/NF‐κB in the P‐group and H‐group on 1 day post‐ICH (*n* = 6, one‐way ANOVA). Data are presented as mean ± SEM; *p* values: ns: *p* > 0.05, *p < 0.05, **p < 0.01, ***p < 0.001.
**Figure S9:** Detailed Methods for Neurobehavioral Evaluation.
1. modified Neurological Severity Score (mNSS).
Neurological deficit scores were independently assessed by two investigators blinded to the experimental grouping. The modified Neurological Severity Score (mNSS) was used to evaluate neurological injury, a comprehensive scale including tests of motor function, sensory response, reflex integrity, and balance ability. Scores ranged from 0 to 18: 13–18 indicated severe injury, 7–12 moderate injury, and 1–6 mild injury.
2. Open Field Test.
Locomotor activity was evaluated using the open field test. The apparatus was a 100 cm × 100 cm × 40 cm arena. Each rat was gently placed in the center and allowed to freely explore for 5 min, with movement recorded continuously. The arena was thoroughly cleaned between tests to eliminate residual odors. Behavioral data were analyzed by two independent investigators using ViewPoint behavior analysis software. Total moving distance and average velocity were quantified.
3. Morris Water Maze (MWM).
Spatial learning and memory were assessed using the Morris Water Maze starting at 7 days after ICH, according to standard protocols. Six rats per group were trained for 5 consecutive days (4 trials per day, maximum 60 s per trial). A probe trial (60 s) was performed on day 6 with the platform removed. Escape latency was recorded during training. The number of platform crossings and time spent in the target quadrant were analyzed in the probe test. All behavioral data were collected for subsequent statistical analysis.

**Table S1:** Detailed information of the individuals enrolled in this study.

**Table S2:** Baseline information of the participants in the Olink proteomics analysis.

**Table S3:** Experimental animal allocation.

## Data Availability

The data that support the findings of this study are available from the corresponding author upon reasonable request.
