# Supplementary material for: Olink Proteomics Reveals CCL2 Aggravates Perihematomal Edema After Intracerebral Hemorrhage in High‐Altitude Migrants Via CCR2/NF‐κB‐Mediated Blood–Brain Barrier Disruption
Source: CNS Neurosci Ther. 2026 Apr 7;32(4):e70868. doi: 10.1002/cns.70868 (PMC13055196; doi:10.1002/cns.70868)
Supplement: Supplementary file 1 — Figure S1: Schematic diagram of the experimental design and research methods. P‐group: plain group; H‐group: high‐altitude group; ICH: intracerebral hemorrhage; WB: Western blot; ELISA: enzyme‐linked immunosorbent assay; IF: frozen section immunofluorescence; TEM: transmission electron microscopy; MWM: Morris water maze test; BWC: brain water content; mNSS: modified neurological severity score; EB: Evans blue; CCL2‐INT‐AAV: adeno‐associated virus (AAV) vector expressing CCL2‐targeted shRNA; r‐CCL2: recombinant CCL2 protein; CCR2‐I: CCR2‐specific inhibitor; CCL2‐Ab: CCL2 neutralizing antibody; BSA: bovine serum albumin; DMSO: dimethyl sulfoxide. Figure S2: Cerebral edema and neurobehavioral changes post‐ICH in P‐group vs. H‐group rats. A. Statistical analysis of brain water content in P‐group and H‐group rats at 1d, 3d, and 7d post‐ICH (n = 6; two‐way ANOVA (Bonferroni post hoc)). B. Trends and intergroup comparisons of modified Neurological Severity Score (mNSS) between the P‐group and the H‐group rats at various time points post‐ICH (n = 6; repeated‐measures two‐way ANOVA with Bonferroni post hoc test). C. Changes in 5‐min locomotor distance and intergroup comparisons in the open field test of rats in the P‐group and H‐group at 1d, 3d, and 7d post‐ICH (n = 6; repeated‐measures two‐way ANOVA). D. Representative images of the open field test between the P‐group and the H‐group rats at 1d, 3d, and 7d post‐ICH. E. Morris water maze (MWM) trajectories: Upper panels (Learning phase) and lower panels (Memory phase) show paths of P‐sham, P‐ICH‐7d, H‐sham, H‐ICH‐7d rats. F. Statistical analysis of MWM test results in P‐group and H‐group rats on day 7 post‐ICH (platform crossovers, escape latency, target quadrant time, average speed) (n = 6, one‐way ANOVA); G. Representative images of BBB ultrastructure (TEM, scale bar = 0.5 μm) in the P‐group and H‐group rats at 1d, 3d, 7d post‐ICH; yellow arrows mark BBB structure. H. Representative MRI (T2) images of P‐group and H‐group r [file CNS-32-e70868-s003.docx]

**
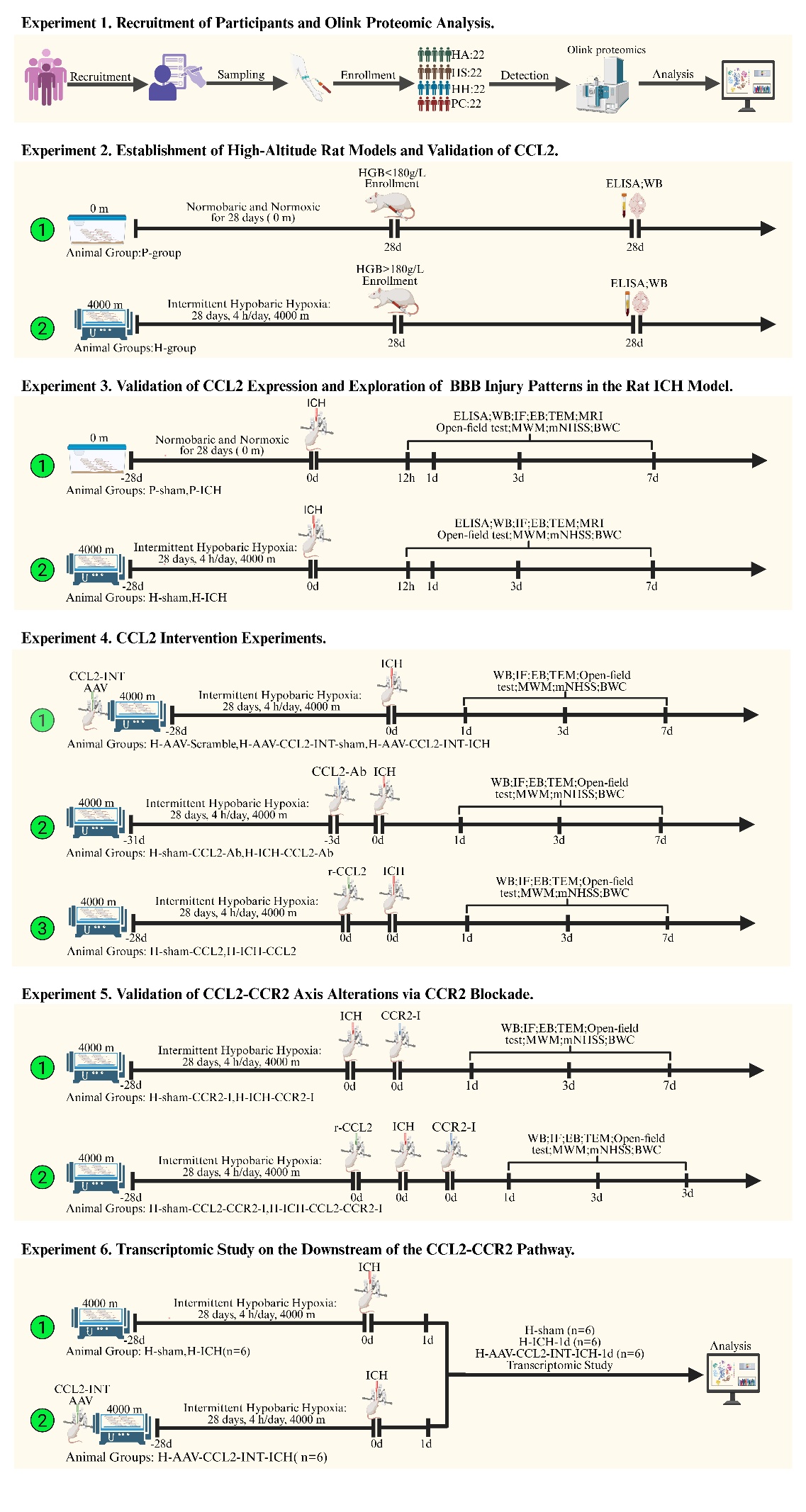
**

**Supplemental Figure 1. Schematic diagram of the experimental design and research methods.** P-group: plain group; H-group: high-altitude group; ICH: intracerebral hemorrhage; WB: Western blot; ELISA: enzyme-linked immunosorbent assay; IF: frozen section immunofluorescence; TEM: transmission electron microscopy; MWM: Morris water maze test; BWC: brain water content; mNSS: modified neurological severity score; EB: Evans blue; CCL2-INT-AAV: adeno-associated virus (AAV) vector expressing CCL2-targeted shRNA; r-CCL2: recombinant CCL2 protein; CCR2-I: CCR2-specific inhibitor; CCL2-Ab: CCL2 neutralizing antibody; BSA: bovine serum albumin; DMSO: dimethyl sulfoxide.


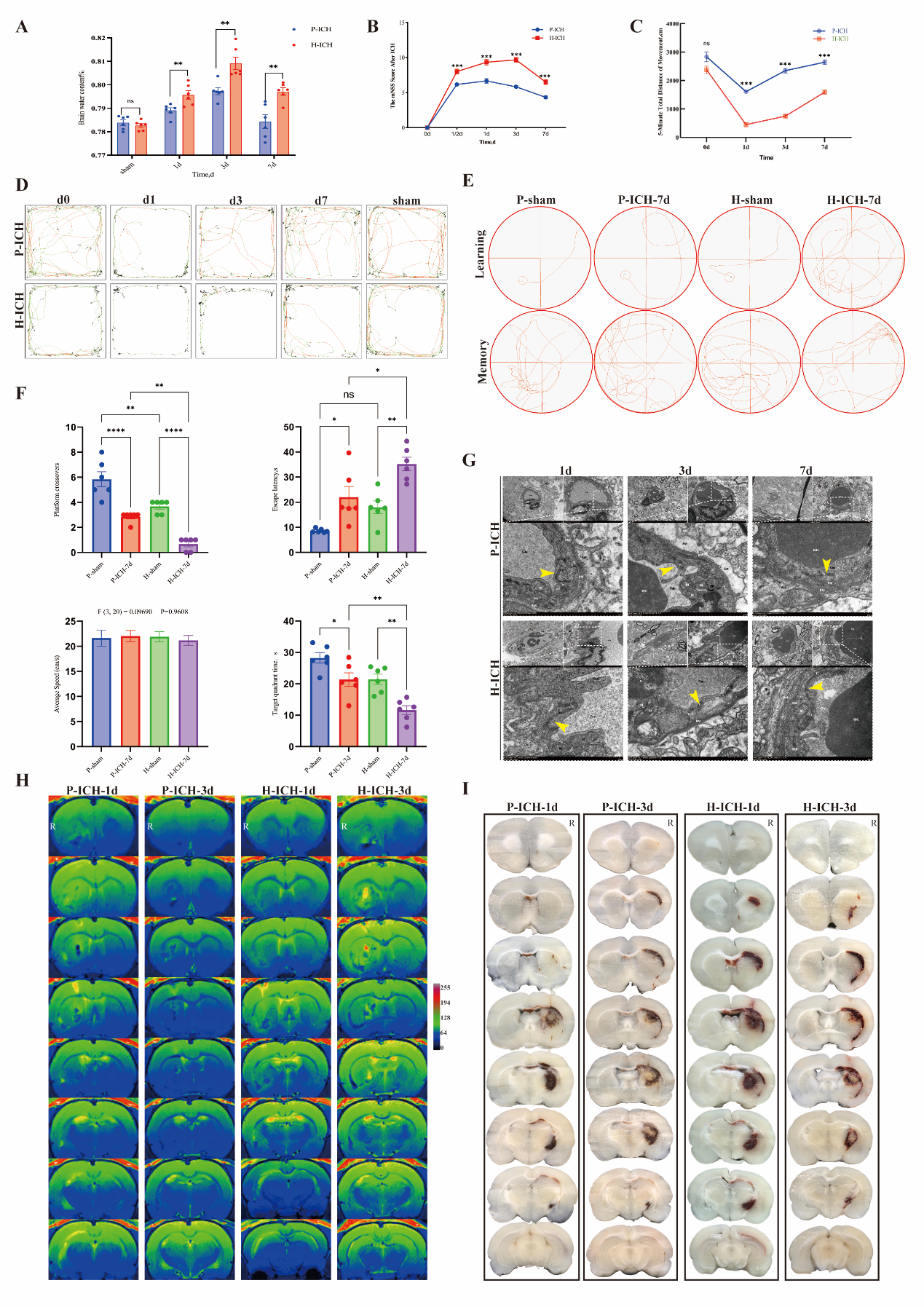


**Supplemental Figure 2. Cerebral edema and neurobehavioral changes post-ICH in P-group vs. H-group rats. A**. Statistical analysis of brain water content in P-group and H-group rats at 1d, 3d, and 7d post-ICH (n=6; two-way ANOVA (Bonferroni post-hoc)). **B**. Trends and intergroup comparisons of modified Neurological Severity Score (mNSS) between the P-group and the H-group rats at various time points post-ICH (n=6; repeated-measures two-way ANOVA with Bonferroni post hoc test). **C**. Changes in 5-minute locomotor distance and intergroup comparisons in the open field test of rats in the P-group and H-group at 1d, 3d, and 7d post-ICH (n=6; repeated-measures two-way ANOVA). **D**. Representative images of the open field test between the P-group and the H-group rats at 1d, 3d, and 7d post-ICH. **E**. Morris water maze (MWM) trajectories: Upper panels (Learning phase) and lower panels (Memory phase) show paths of P-sham, P-ICH-7d, H-sham, H-ICH-7d rats. **F**. Statistical analysis of MWM test results in P-group and H-group rats on day 7 post-ICH (platform crossovers, escape latency, target quadrant time, average speed) (n=6, one-way ANOVA); **G**. Representative images of BBB ultrastructure (TEM, scale bar=0.5 μm) in the P-group and H-group rats at 1d, 3d, 7d post-ICH; yellow arrows mark BBB structure. **H**. Representative MRI(T2) images of P-group and H-group rats at 1d and 3d post-ICH. **I**. Representative images of pathological sections from P-group and H-group rats at 1d and 3d post-ICH. Data are presented as mean ± SEM; P values: ns: *P* > 0.05, **P* < 0.05, ***P*< 0.01, ****P*< 0.001.

**
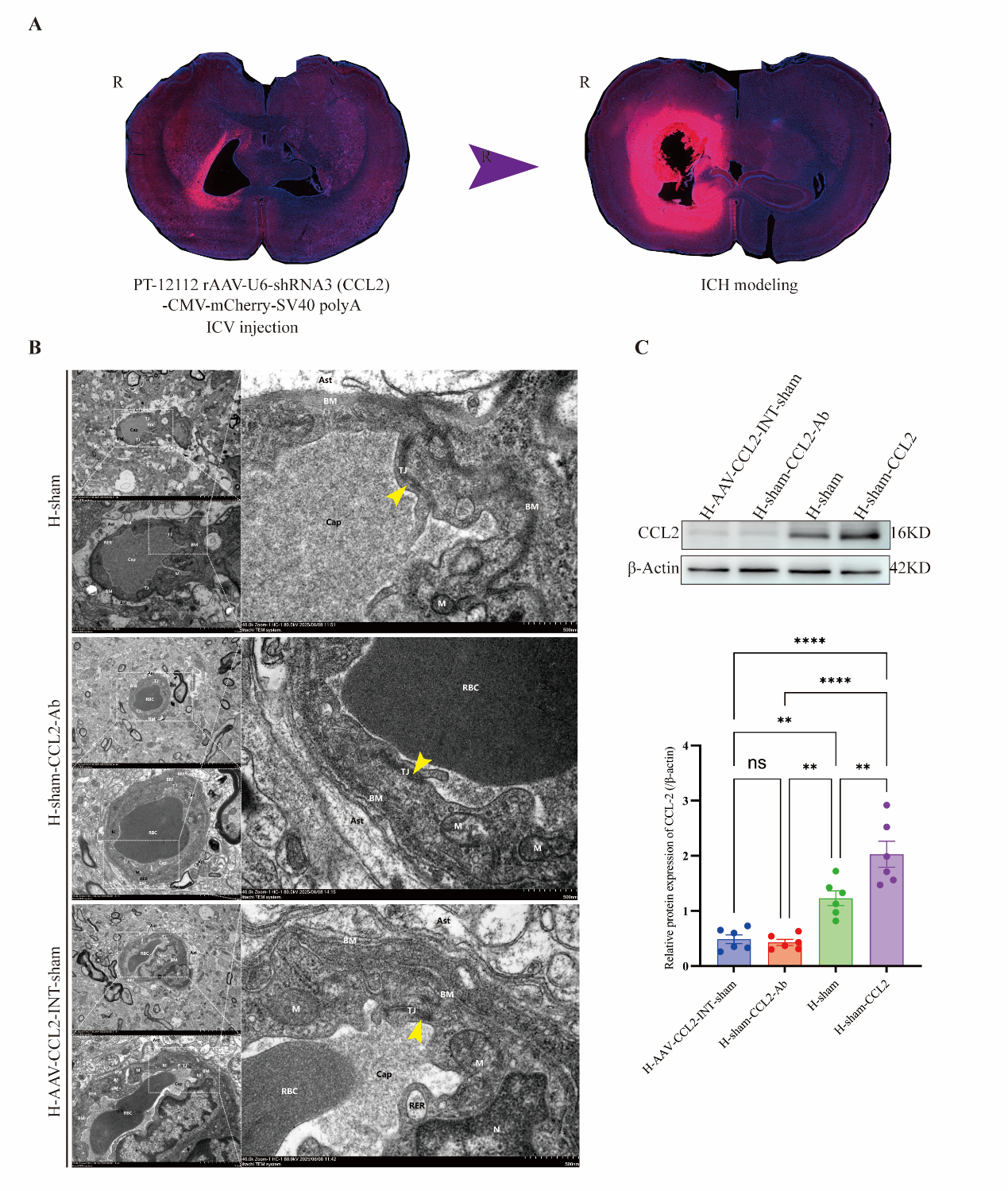
**

**Supplemental Figure 3. Effects of CCL2 intervention on CCL2 expression and blood-brain barrier ultrastructure in sham-operated rats.** **A**. The expression of the virus in the H-AAV-CCL2-INT group after intracerebroventricular injection of AAV virus and subsequent intracerebral hemorrhage (ICH) modeling is shown in the figure, indicated by the red fluorescence inherent to the virus. **B**. TEM images of BBB ultrastructure in sham-operated rats from each group: H-sham, H-CCL2 Ab-sham, and H-AAV-CCL2-INT-sham. Yellow arrows indicate tight junction structures (T), showing differences in the integrity of interendothelial tight junctions across intervention groups. **C.** WB analysis and quantitative comparison of relative CCL2 protein expression (normalized to β-Actin) in brain tissues of sham-operated rats from each group. The highest CCL2 expression was observed in the H-sham-CCL2 group, while the lowest expression was detected in the H-AAV-CCL2-INT-sham group, and expression was significantly downregulated in the H-sham-CCL2 Ab group. Data are presented as mean ± SEM; P values: ns: *P* > 0.05, **P* < 0.05, ***P*< 0.01, ****P*< 0.001.

**
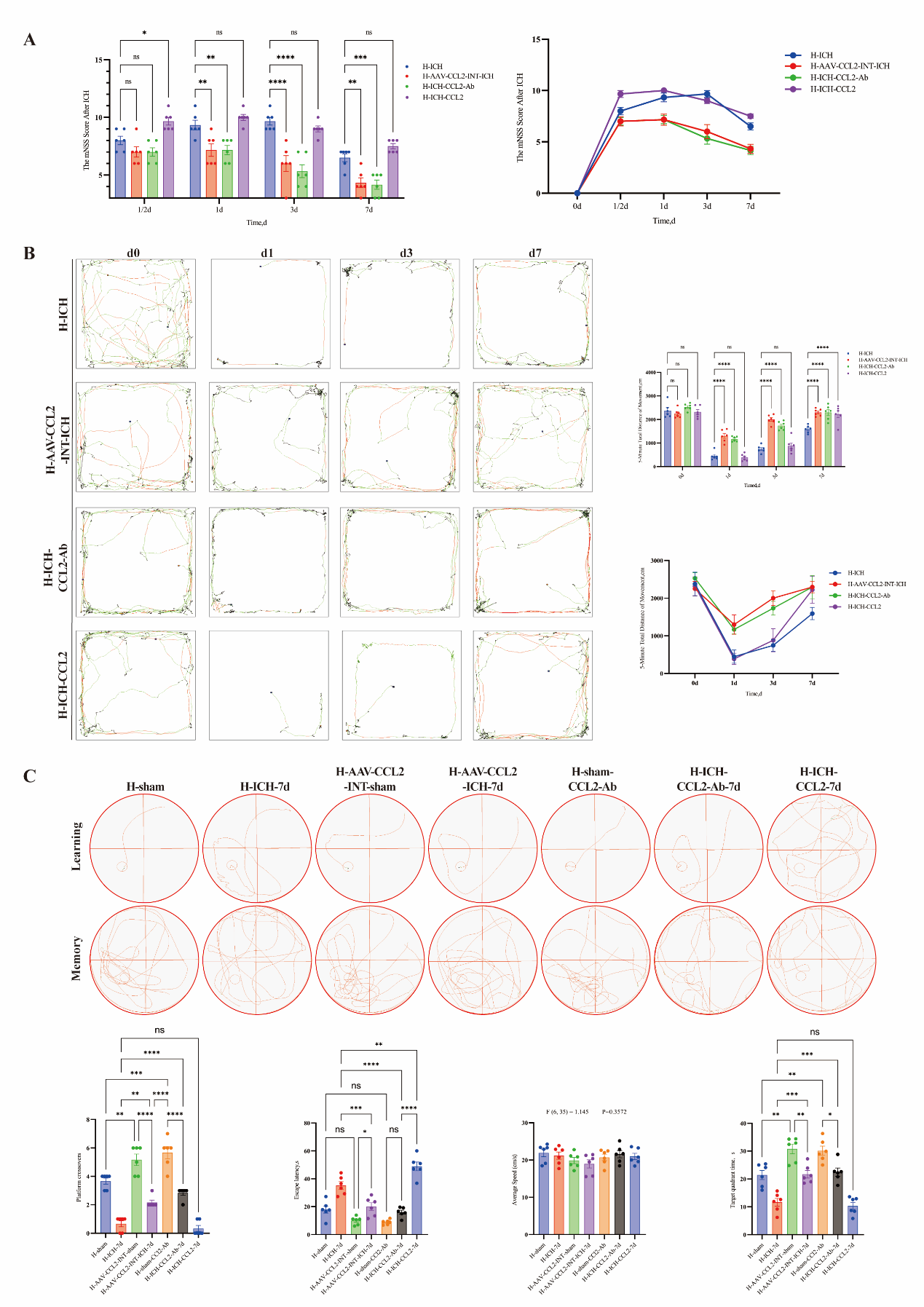
**

**Supplemental Figure 4. Neurobehavioral changes in each group after CCL2 intervention.** **A**. Bar graph and line graph of the results of the statistical analysis of the mNSS in each group after CCL2 intervention (n = 6; repeated-measures two-way ANOVA). **B**. Activity trajectory plots of the 5-minute open field test in each group after CCL2 intervention. Bar graph and line graph of the statistical analysis of the 5-minute total distance traveled in the open field test in each group after CCL2 intervention (n = 6; two-way ANOVA). **C**. Trajectory plots and statistical analysis of the results of the Morris water maze test for each group after CCL2 intervention (n = 6; one-way ANOVA). Data are presented as mean ± SEM; P values: ns: *P* > 0.05, **P* < 0.05, ***P*< 0.01, ****P*< 0.001.

**
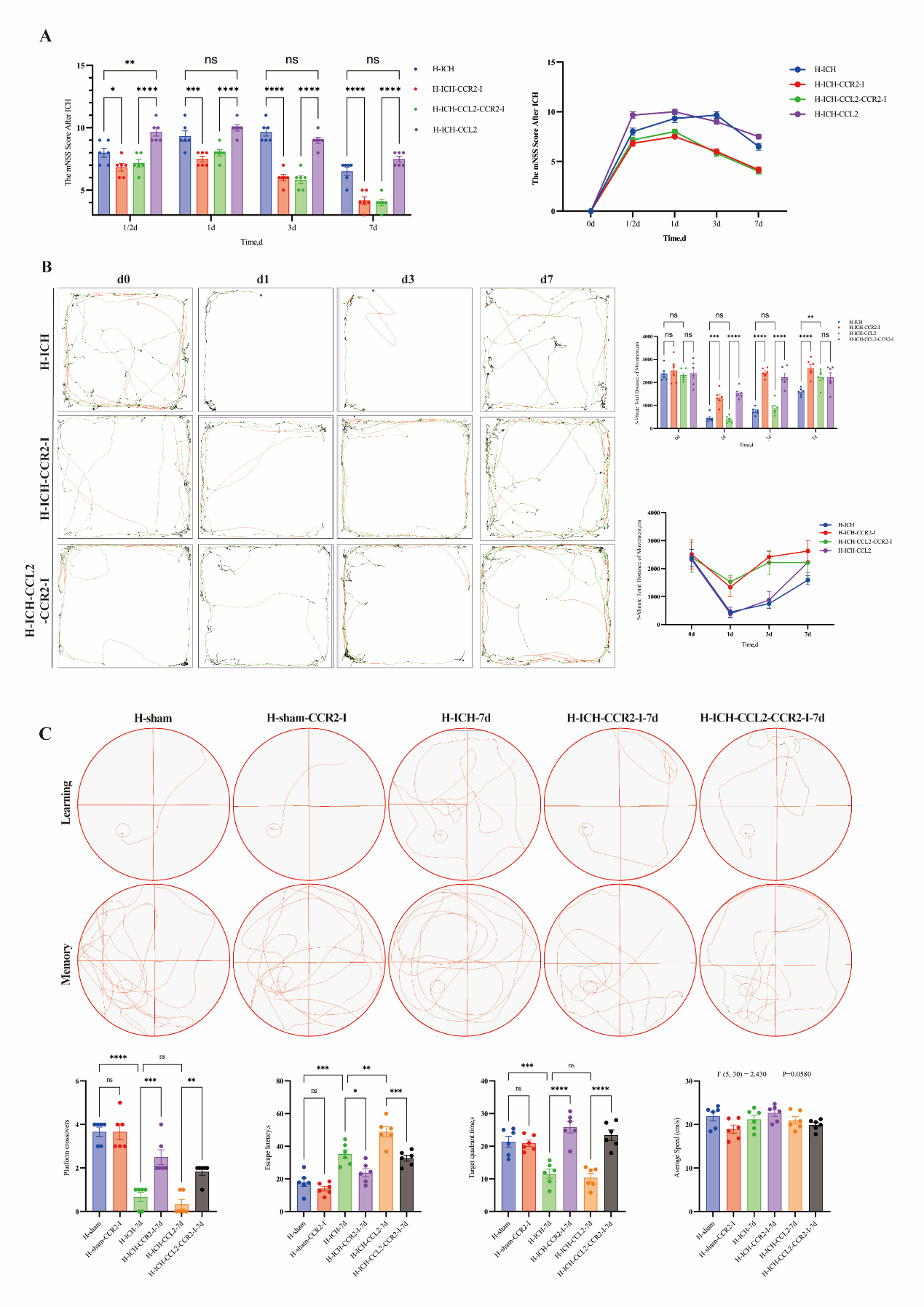
**

**Supplemental Figure 5. Changes in neurobehavior after blocking CCR2.** **A**. Bar graph and line graph showing the results of the statistical analysis of the mNSS for each group following CCR2 blockade (n=6; repeated-measures two-way ANOVA). **B**. Five-minute activity trajectory plots from the open field test for each group following CCR2 blockade. Bar graph and line graph showing the results of the statistical analysis of the 5-minute total distance traveled in the open field test for each group following CCR2 blockade (n=6; two-way ANOVA). **C**. Morris water maze test results. Representative trajectory plots of the training phase and probe trial of the MWM test in each group on day 7 after ICH following CCR2 blockade are shown. Statistical analysis of escape latency, number of platform crossings, time spent in the target quadrant, and average speed (n=6; one-way ANOVA); Data are presented as mean ± SEM; P values: ns: *P* > 0.05, **P* < 0.05, ***P*< 0.01, ****P*< 0.001.

**
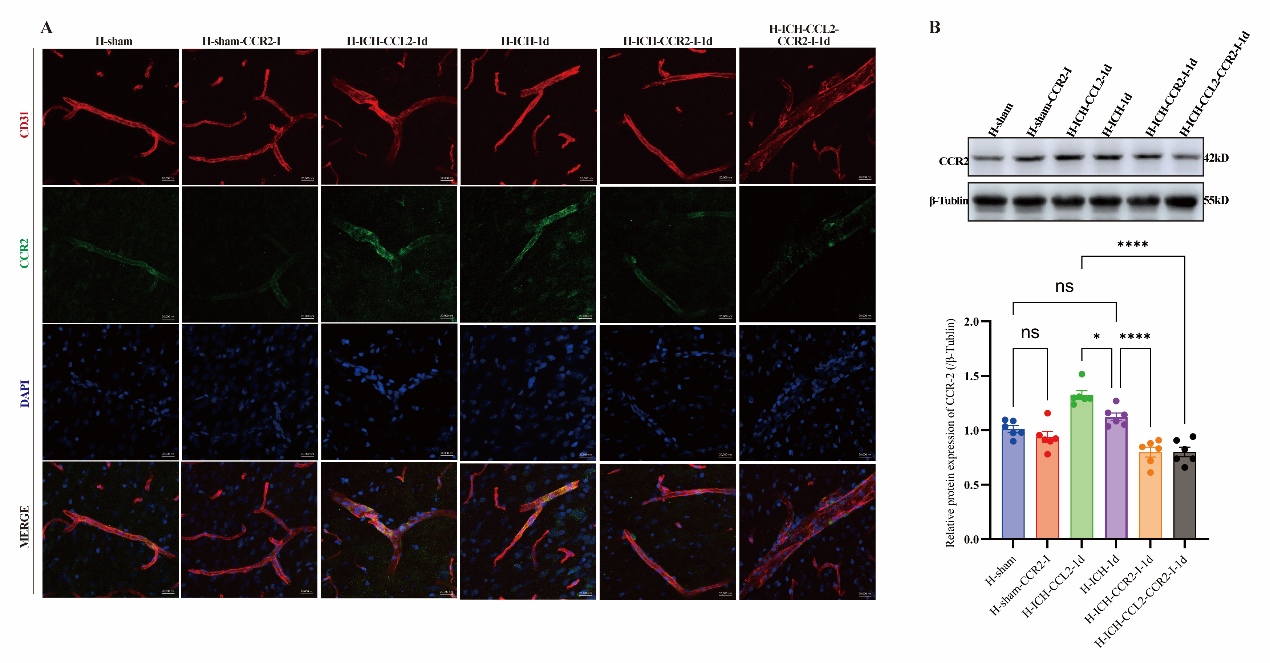
**

**Supplemental Figure 6. Changes in CCR2 expression following CCR2 blockade. A.** Representative photomicrographs of CCR2 expression detected by immunofluorescence staining in each group at 1 day post-ICH following CCR2 blockade (scale bar=50 μm): CCR2 (green), CD31 (red), DAPI (blue). **B**. Representative blots and statistical analysis of CCR2 protein expression levels (normalized to β-Tubulin) in perihematomal brain tissue from each group 1 day post-ICH after CCR2 blockade (WB, n = 6; one-way ANOVA). Data are presented as mean ± SEM; P values: ns: *P*>0.05, **P*<0.05, ***P*<0.01, ****P*<0.001.

**
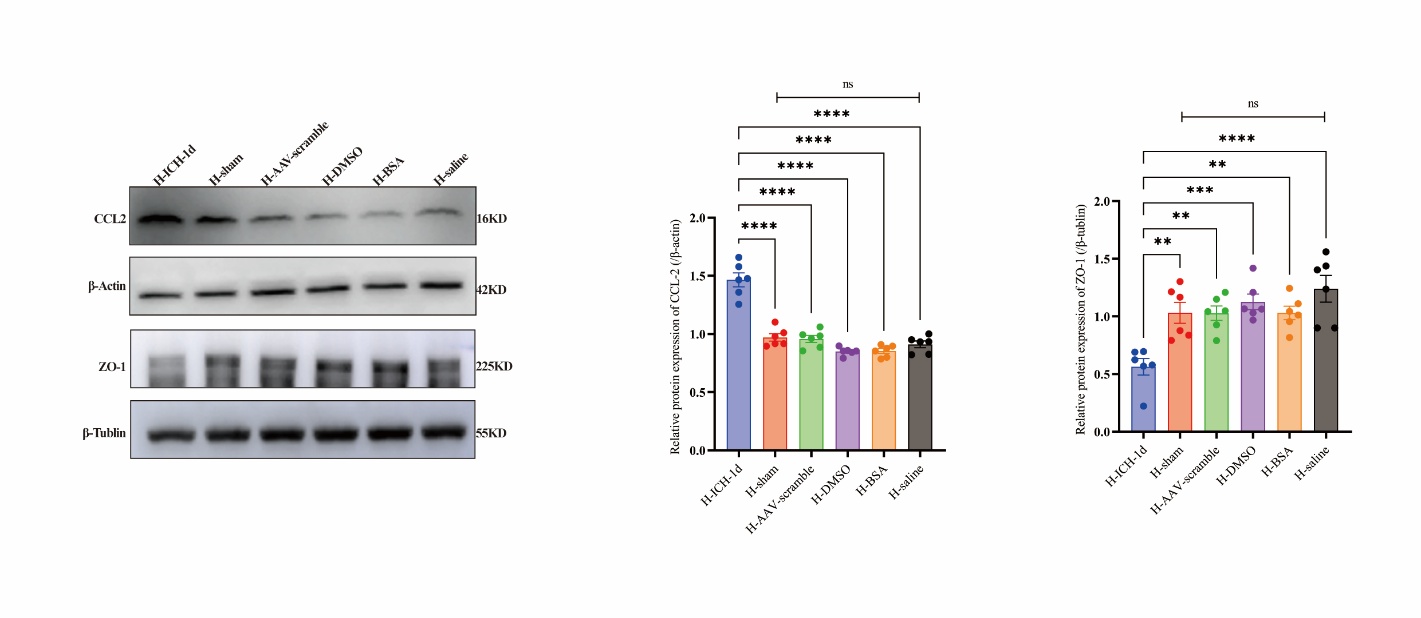
**

**Supplemental Figure 7. Effects of different solvents on CCL2 and ZO-1.** WB analysis of CCL2 and ZO-1 in different solvents groups rats: Blots: CCL2 + β-actin; ZO-1 + β-Tubulin for groups in this panel. Quantification: Relative protein levels (n=6; one-way ANOVA). Data are presented as mean ± SEM; P values: ns: *P* > 0.05, **P* < 0.05, ***P*< 0.01, ****P*< 0.001.

**
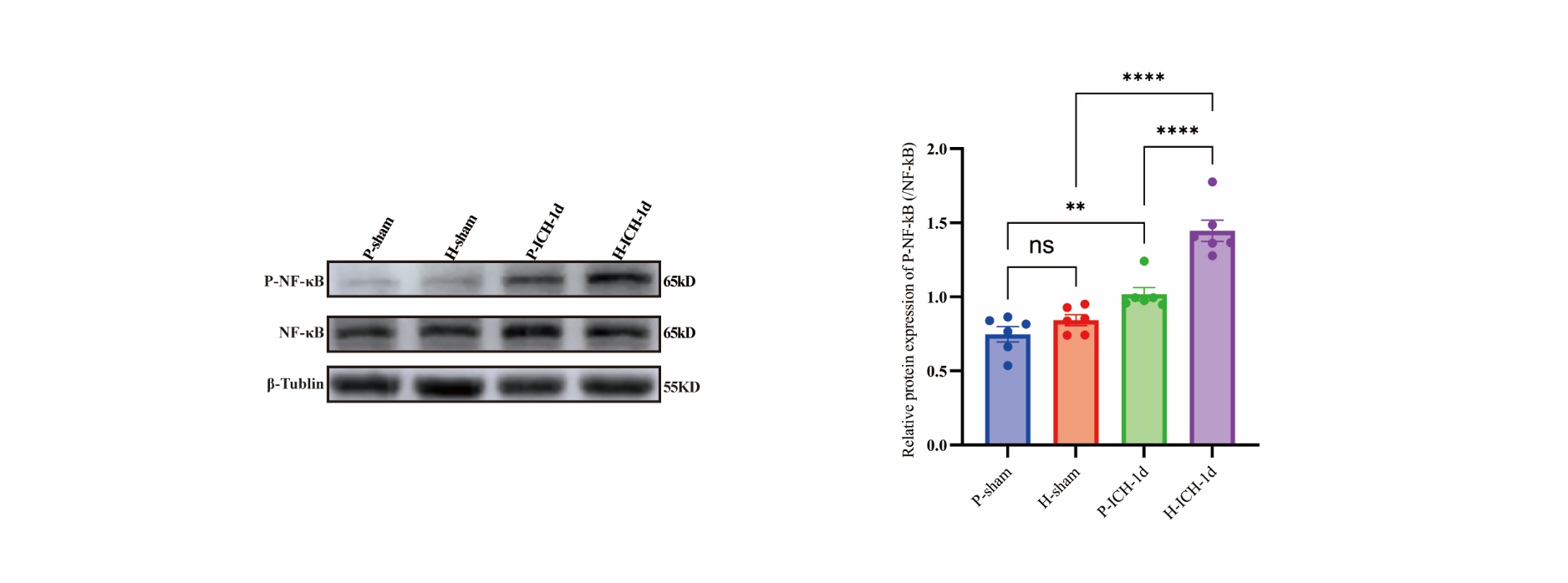
**

**Supplemental Figure 8.** WB images and statistical analysis of the relative gray values of P-NF-κB/NF-κB in the P-group and H-group on 1 day post-ICH (n=6, one-way ANOVA). Data are presented as mean ± SEM; P values: ns: *P* > 0.05, **P* < 0.05, ***P*< 0.01, ****P*< 0.001.

**Supplementary Material 9**: Neurobehavioral Test Methods

**1) modified Neurological Severity Score (mNSS)**

Neurological deficit scores were independently assessed by two investigators blinded to the experimental grouping. The modified Neurological Severity Score (mNSS) was used to evaluate neurological injury, a comprehensive scale including tests of motor function, sensory response, reflex integrity, and balance ability. Scores ranged from 0 to 18: 13–18 indicated severe injury, 7–12 moderate injury, and 1–6 mild injury.

**2) Open Field Test**

Locomotor activity was evaluated using the open field test. The apparatus was a 100 cm × 100 cm × 40 cm arena. Each rat was gently placed in the center and allowed to freely explore for 5 minutes, with movement recorded continuously. The arena was thoroughly cleaned between tests to eliminate residual odors. Behavioral data were analyzed by two independent investigators using ViewPoint behavior analysis software. Total moving distance and average velocity were quantified.

**3) Morris Water Maze (MWM)**

Spatial learning and memory were assessed using the Morris Water Maze starting at 7 days after ICH, according to standard protocols. Six rats per group were trained for 5 consecutive days (4 trials per day, maximum 60 s per trial). A probe trial (60 s) was performed on day 6 with the platform removed. Escape latency was recorded during training. The number of platform crossings and time spent in the target quadrant were analyzed in the probe test. All behavioral data were collected for subsequent statistical analysis.
